# *ATG7*-deficient fibroblast promotes breast cancer progression via exosome-mediated downregulation of *SCARB1*

**DOI:** 10.1038/s41419-025-07885-6

**Published:** 2025-07-24

**Authors:** Kangdi Li, Ting Liu, Zhihong Luo, You Yu, Yi Liu, Zhaoqing Zhang, Wenhua Li

**Affiliations:** 1 Hubei Key Laboratory of Cell Homeostasis, College of Life Sciences, Wuhan University, 430072, Wuhan, People’s Republic of China; 2 Wuhan University Shenzhen Research Institute, 518057, Shenzhen, People’s Republic of China; 3 The Central Hospital of Wuhan, Tongji Medical College, Huazhong University of Science and Technology, 430014, Wuhan, People’s Republic of China; 4 Department of Pain Rehabilitation, Tongren Hospital of Wuhan University (Wuhan Third Hospital), 430065, Wuhan, People’s Republic of China

**Keywords:** Cancer microenvironment, Breast cancer, Cell invasion

## Abstract

Although autophagy-related gene 7 (*ATG7*) acts as an E1-like activating enzyme and is essential for autophagy, it frequently performs broader roles involved in the modulation of diverse signaling pathways that affect cell proliferation, survival, migration and transformation. *ATG7* is often downregulated in various cancers. However, the role of *ATG7* in fibroblasts in regulating breast carcinoma remains poorly understood. Herein, we revealed that aberrantly low expression of *ATG7* in breast stroma is clinically relevant to breast cancer progression. Loss of *ATG7* expression results in fibroblasts acquiring the hallmarks of cancer-associated fibroblasts (CAFs), which finally promote the proliferation, metastasis of breast cancer in vivo and vitro. Detailed regulatory mechanisms showed that *ATG7*-deficient fibroblasts secrete a new miRNA (miR-6803b) and are then transported into breast cancer cells by exosomes. In breast cancer, miR-6803b targets the *SCARB1* gene to inhibit its expression and then promote cancer cell metastasis, resulting in cancer progression. Thus, our results indicate that *ATG7* expression in fibroblasts plays a vital role in regulating breast cancer tumorigenesis and progression by modifying stromal–epithelial crosstalk and remodeling the tumor microenvironment (TME). These results suggest that *ATG7* can function as a tumor suppressor and represent a new candidate for prognosis and targeted therapy.

## Introduction

Breast cancer is the most frequently recognized cancer in females throughout the world and is a major cause of cancer-related deaths [1]. Despite improvements in diagnosis and treatment, the mortality rate of breast cancer remains higher than expected, mainly due to the recurrence and metastasis of cancer cells to vital organs [2].

Breast tumor tissue is highly complex and heterogeneous and mainly consists of neoplastic epithelial cells and the surrounding TME [3]. The TME is a continuously evolving entity during tumor progression encompassing a diverse collection of cells that are recruited to cancer tissues; these cells mainly include stromal cell components, such as fibroblasts, macrophages, adipocytes, endothelial cells, and inflammatory cells, as well as extracellular matrix [4, 5]. Recently, TME has been recognized to play a crucial role in tumorigenesis, relapse and resistance to therapy in breast cancer [6]. The crosstalk between cancer cells and their surrounding stromal components, which involves reciprocal autocrine and paracrine signaling, regulates cell survival, proliferation, metastasis, differentiation, and angiogenesis [7, 8]. This crosstalk also modifies cellular compartments, which results in the coevolution of tumor cells and their microenvironment [9, 10]. CAFs are the largest population of stromal cells within breast cancers, and increasing evidence suggests that these cells enhance tumor initiation, progression, and metastasis by secreting diverse inflammatory cytokines and growth factors and remodeling the extracellular matrix [11, 12].

Genomic instability is one of the most common characteristics of tumors and is a major regulator of tumor adaptation and evolution in response to challenges arising from the TME [13, 14]. This instability leads to increased mutations, somatic copy number alterations, and epigenomic changes, which have classically been considered the underlying mechanism behind tumor initiation, progression, metastasis and therapeutic responses [15]. Computational analysis has shown that breast cancer cells frequently undergo mutations, causing abnormalities in the function and expression of a variety of cancer-driving gene proteins, such as BRCA, TP53, PIK3CA, PTEN, KRAS, and GATA3 [16, 17]. Most of these genes are involved in cell cycle regulation, survival, gene transcription and other cell signaling cascades, influencing tumor cell-autonomous responses and tumor cell-microenvironment interactions [18, 19]. Considerable reports have focused on mutations in the tumor epithelial cells themselves and revealed that genetic changes in tumor epithelial cells drive tumorigenesis and regulate subsequent neoplastic progression and the differentiation of histological subtypes [20]. In fact, genetic instability in breast cancer often leads to mutations or the abnormal expression of cancer-related genes in the stroma, including those in CAFs and macrophages, which are associated with a poor prognosis for patients [21, 22]. However, whether stromal cells harboring mutations or alterations in the expression of these genes can orchestrate the progression of mammary malignancies by crucially modifying stromal–epithelial crosstalk and remodeling the TME remains unclear.

Autophagy-related gene 7 (ATG7) acts as an E1-like activating enzyme and is essential for autophagy and cytoplasmic-to-vacuole transport by facilitating both microtubule-associated protein light chain 3 (LC3)-phosphatidylethanolamine and ATG12 conjugation [23]. In addition, ATG7 also performs broader roles beyond autophagy and is involved in the modulation of diverse signaling pathways that affect cell survival, cell cycle progression, gene expression, protein secretion, cell polarity, migration, and cell transformation [24]. ATG7 coordinates the p53-mediated cell division cycle and cell apoptosis via physical interaction with p53 during metabolic stress [25]. The antiviral activity of IFN-γ in macrophages requires ATG7 but not the induction of autophagy [26]. Evidence also exists for the direct involvement of ATG7 proteins in lipid droplet formation and the localization of secretory lysosomes within the actin ring [24, 27]. However, the effects of *ATG7* gene mutation caused by genetic instability in peritumoral fibroblasts in breast carcinoma on tumorigenesis and progression as well as its functional role remain poorly understood.

In this study, we demonstrate that abnormal *ATG7* expression in fibroblasts promotes breast cancer progression through paracrine signaling. Specifically, a loss of ATG*7* expression stimulates the conversion of fibroblasts into activated fibroblasts and results in the acquisition of characteristic CAFs functions that promote breast cancer epithelial cell proliferation, metastasis and stemness through exosome-mediated miR-6803b. Compared with normal fibroblasts, *ATG7*^−/−^ fibroblasts expressed and secreted more miR-6803b. These miRNAs are shuttled from fibroblasts to breast cancer cells mediated by exosomes and then inhibit their target gene, *SCARB1*, which subsequently leads to breast cancer malignant progression. Our results demonstrate that *ATG7* expression in fibroblasts has a crucial role in modifying stromal–epithelial crosstalk and disrupting homeostasis in the TME and reveal that *ATG7* in fibroblasts and *SCARB1* in breast cancer cells may be new indicators for the diagnosis of breast cancer.

## Materials and methods

### Reagents and antibodies

GW4869 (10 μM, dissolved in DMSO) was purchased from Santa Cruz (sc-218578, CA, USA). Antibodies against ATG7 (A2856, 1:3000) and LC3 (L8918, 1:3000) were purchased from Sigma Aldrich. Antibodies against ACTA2 (14395-1-AP, 1:3000), TSG101 (14497-1-AP, 1:1000), Snail (13099-1-AP, 1:3000), CD9 (20597-1-AP, 1:2000) and Occludin (27260-1-AP, 1:1000) were purchased from Proteintech. The antibody for Scarb1 (ab217318, 1:1000) was purchased from Abcam. Antibodies against FAP (#52818, 1:3000), PDGFRα (#3174, 1:3000), E-cadherin (#3195, 1:3000) and Vimentin (#5741, 1:3000) were purchased from Cell Signaling Technology. Antibodies against Rab27a (A1934, 1:1000) and GAPDH (AC002, 1:100000) were purchased from ABclonal Technology.

### Cell culture

The human breast cancer cell lines MDA-MB-231, MDA-MB-468 and T47D were purchased from ATCC (Manassas, VA, USA). The mouse breast cancer cell line 4T1 and human fibroblasts HFF-1 were purchased from CCTCC (Wuhan, China). WT MEFs were isolated and immortalized from BALB/c mice. *Atg7*^−/−^ MEFs were obtained by WT MEF knockout of the *Atg7* gene which was kindly gifted from Dr. Masaaki Komatsu [28]. All the cell lines were authenticated (STR profiling) and tested for mycoplasma contamination. These cells were cultured in Dulbecco’s modified Eagle’s medium (DMEM) supplemented with 10% fetal bovine serum (HyClone) and 1% penicillin–streptomycin (Beyotime) in a humidified incubator containing 5% CO_2_ at 37 °C.

### Contractility assay

A fibroblast contractility assay was conducted as previously described [29]. Specifically, fibroblasts were washed with PBS to remove trypsin. For a 24-well plate culture system, 5 × 10^4^ cells were resuspended in 400 μL of culture medium, add 150 μL of collagen and 7 μL of 1 M NaOH then mix well. Add 500 μL of the mixture to the 24-well plate, three replicates per group. After 20 min at room temperature, add 600 μL of culture medium, gently scrape the edge of the gel from the wall with a pipette tip, and place the plate in an incubator. Observations were made at specific time points.

### Cell proliferation assay and colony formation assay

For the cell proliferation assays, cells were plated in 96-well plates and incubated with CM, and the total number of cells was counted at specific time points.

For the colony formation assays, colonies were measured by crystal violet (C6158, Sigma‒Aldrich) staining as previously described [29].

### RNA extraction and Q‒PCR

Total RNA was extracted from cultured cells and exosomes using TRIzol (15596026, Invitrogen) according to the manufacturer’s instructions. Reverse transcription was performed using the PrimeScript RT reagent Kit (RR047Q, Takara). Q-PCR was conducted using iTaq Universal Supermixes (1708882, Bio-Rad) and performed on an ABI 7500 system. GAPDH and 18S rRNA were used as reference controls for mRNA and miRNA, respectively. Analysis was performed as previously described [30]. The sequences of all indicated primers are listed in the Supplementary Table 1.

### Isolation and analysis of exosomes

The isolation, electron microscopy (EM) observation, internalization and nanoparticle analysis of exosomes were performed as we described previously [29].

### Exosomal miRNA Distribution Assay

DiD-labeled MEFs cultured in 0.4 μm Transwell inserts were transfected with Cy3-labeled miR-6803b. 6 h later, the cells were washed with DMEM to remove free miRNA and then co-cultured with 4T1 cells seeded on coverslips in 12-well plates for 24 h. Subsequently, 4T1 cells were stained with DAPI (D9542, Sigma). The distribution of exosomal miRNA in 4T1 cells was determined by confocal microscopy (SP8, Leica, Wetzlar, Germany).

### Conditioned medium preparation

The same number of cells was cultured with complete medium for 2 days until the supernatants were collected and centrifuged at 4 °C and 300 g for 10 min, then at 2000*g* for 10 min and at 10,000*g* for 30 min. Finally, the supernatants were filtered through a 0.22-μm filter and mixed with complete medium at a 1:1 ratio.

### Sphere formation assay

Cells were suspended in CM mixed with B-27™ Supplement (12587010, Gibco) and seeded into ultralow attachment 6-well plates (3471, Corning) at a density of 5 × 10^3^ cells/well. After 5 days of culture, spheres larger than 50 μm were photographed and counted.

### Matrigel three-dimensional cultures

The assay was performed as described previously [31].

### Bioinformatics analysis

Gene expression profiles (GEO Accession number: GSE100534, GSE27018, GSE29270, GSE21422) were extracted from the GEO database (https://www.ncbi.nlm.nih.gov/geo/) based on the following criteria: containing normal breast stroma or fibroblasts collected from breast cancer patients who had never undergone any radiotherapy, chemotherapy, or targeted therapy. Then, a scatter plot was generated by GraphPad Prism according to the expression levels of *ATG7* and *SCARB1*. The survival analysis of *ATG7* and *SCARB1* in breast cancer was conducted by the online exploration tool XENA (https://xenabrowser.net/) based on TCGA (BRCA) data and the Kaplan‒Meier plotter online web tool (https://kmplot.com/analysis/).

### Tissue microarray and IHC

The human breast cancer tissue microarray chip was purchased from (Cat# HBreD140Su03) Shanghai Outdo Biotechnology Co. For immunohistochemistry, the microarray chip was stained with primary antibodies overnight at 4 °C, followed by incubation with secondary antibodies at room temperature for 2 h. The level of ATG7 in stroma and epithelium was evaluated by the IHC score ranging from 0 to 12 respectively, which was calculated by multiplying the proportion and intensity score. Two independent experts were blinded to assess each result. Staining intensity was scored as follows: 0 = no staining, 1 = weak staining, 2 =moderate staining, 3 = strong staining. The proportion of positively stained tumor cells was scored as follows: 1 (<10%), 2 (10–50%), 3 (50–75%), and 4 (>75%). The survival analysis of ATG7 in breast cancer was conducted by the Kaplan‒Meier plotter package of R tools.

### Isolation and culture of human breast fibroblasts

We randomly collected samples that meet the following criteria, female breast cancer patients aged 45-75 years with a newly diagnosed, biopsy-proven invasive ductal carcinoma at stage II to III, and had never undergone any radiotherapy, chemotherapy, or targeted therapy, excluding samples that are necrotic or have insufficient tumor content. The isolation and culture of human breast fibroblasts were performed as previously described [32]. Informed consent was obtained from all examined subjects, and the related studies were approved by the ethics committees of the participating hospitals and institutes.

### Animal experiments

An orthotopic mouse model of spontaneous breast cancer metastasis was used in animal experiments. Specifically, a total of 1.5 × 10^6^ WT MEFs or *ATG7*^−/−^ MEFs were mixed with 1 × 10^6^ MDA-MB-231 or 5 × 10^5^ 4T1 cells that had been engineered to stably express firefly luciferase and suspended in 100 μL of Matrigel (356230, Biocoat)/PBS mixture, then these cells were randomly injected subcutaneously into the left fourth mammary fat pad of five 6-week-old female BALB/C-NU or BALB/C mice. For bioluminescence imaging, mice were anesthetized and abdominally injected with 200 mg/g of d-luciferin (40902ES01, Yeasen) in PBS. 15 min later, bioluminescent imaging system (Lumina II, Caliper Life Science) was used to detect tumor size and metastasis. All measurements were in a blinded manner. Mice were handled according to the Guidelines of the China Animal Welfare Legislation, as provided by the Committee on Ethics in the Care and Use of Laboratory Animals of Wuhan University. The experimental protocols were approved by the Experimental Animal Centre of Wuhan University.

### Statistical analysis

All data were obtained from at least three independently repeated experiments. Data analysis was performed using Microsoft Excel and GraphPad Prism 7. All quantitative data are presented as the mean ± SD. Except for the data on comparison of ATG7 staining IHC scores were determined by Mann–Whitney test, and all other quantitative significance between two independent groups was determined by Student’s *t* test, unless otherwise stated. *P* < 0.05 was considered statistically significant. *P < 0.05, **P < 0.01, ***P < 0.001, ns, not significant.

## Results

### Aberrantly low expression of *ATG7* in the fibroblasts is associated with breast cancer progression

**Fig. 1:**
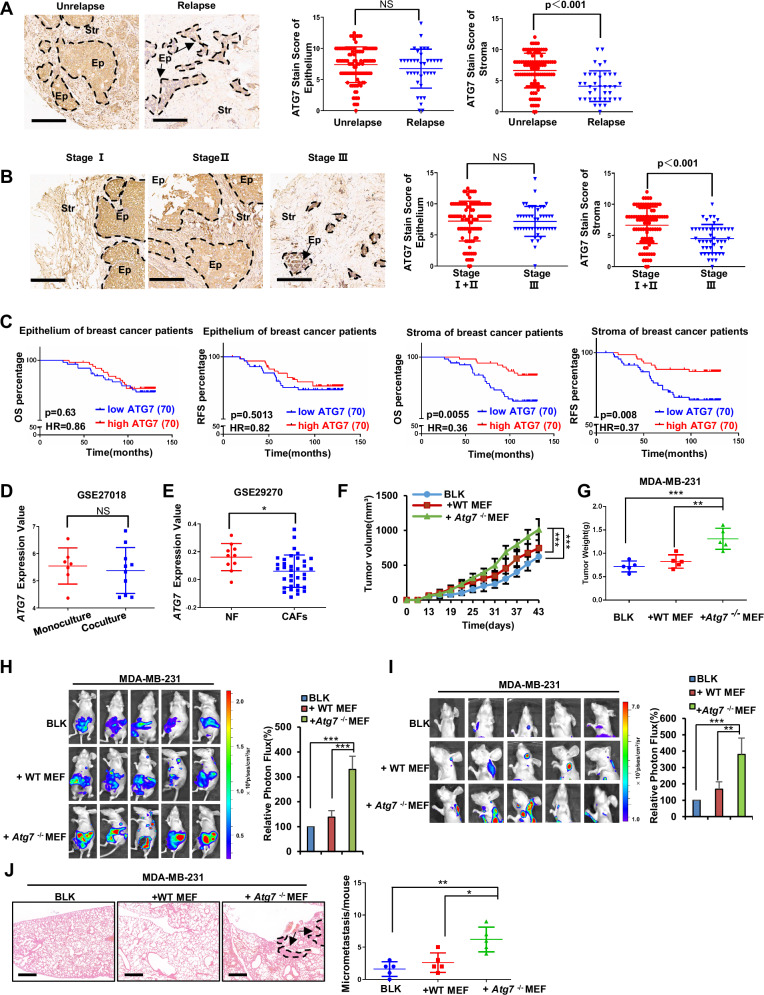
Aberrantly low expression of *ATG7* in the fibroblasts is associated with breast cancer progression. **A**, **B** Left: TMA analysis of 140 breast cancer samples for the expression of ATG7. Representative images are shown. Scale bar = 200 μm; Right: Scatterplots show the score of ATG7 staining intensity in the epithelial part (identified by glandular or nest structures, distinct borders, and round to oval dark-staining nuclei) or stromal part (identified by loosely arranged spindle-shaped fibroblasts with smaller, more uniform, and less hyperchromatic nuclei) among patients with relapse (**A**) or different TNM stages (**B**). **C** Kaplan‒Meier survival analysis of the relationship between the OS, RFS and the expression of *ATG7* based on the TMA (by log-rank). The ATG7-low and ATG7-high groups are defined based on the median IHC-score. **D** *ATG7* expression levels in breast cancer cells that were monocultured or cocultured with fibroblasts based on the GEO database (GSE27018). The measure unit of expression is log2-transformed normalized expression levels from the microarray results. **E** *ATG7* expression levels in NF (Normal mammary fibroblasts from healthy individuals who underwent breast reduction surgery) and CAFs from the GEO database (GSE29270). The measure unit of expression is log2-transformed normalized expression levels from the microarray results. **F** Xenograft assays of MDA-MB-231-Luc cells mixed with the indicated MEFs. Tumor growth curves are shown. **G** Scatterplots of the individual weights of tumors. **H** Xenograft tumors of MDA-MB-231-Luc cells were quantified using bioluminescence imaging. Representative images are shown. **I** Metastatic colonization of MDA-MB-231-Luc cells was quantified using bioluminescence imaging. Representative images are shown. **J** Left: Representative H&E-stained sections of lungs in MDA-MB-231-Luc xenograft mice; Arrows and dotted lines indicate metastatic colonization in lung sections, characterized by dense clusters of atypical tumor cells, disrupted alveolar structure, and nuclear pleomorphism. Right: Quantification of metastatic colonization in the lungs. The data of panel (**A**, **B**) were analyzed by Mann–Whitney test, other data are presented as mean ± s.d. of three independent experiments and analyzed by Student’s *t* test. NS not significant, *p < 0.05; **p < 0.01; ***p < 0.001.

Emerging evidence underscores the dual role of *ATG7* as both an oncogene and a tumor suppressor, orchestrating a multifaceted impact on cellular processes encompassing cell proliferation, survival, traffic, protein secretion, signaling transduction, and cancer treatment [33]. To determine the role of ATG7 in breast cancer progression, we assembled a cohort of 140 primary breast cancer tumors, spanning diverse TNM stages, a standardized classification system used to assess cancer progression based on tumor size (T), lymph node involvement (N), and distant metastasis (M) [34]. After demonstrating the specificity of ATG7 antibody in immunohistochemistry (Supplementary Fig. 1A), we examined *ATG7* expression in different breast cancer patient samples by immunohistochemistry analysis using staining intensity score (Supplementary Fig. 1B). Remarkably, the anomalous downregulation of ATG7 in relapsed breast cancer patients was predominantly observed in the stromal region as opposed to the epithelium (Fig. 1A). In addition, advanced TNM stage breast cancer patients exhibited lower ATG7 protein expression in the stroma (Fig. 1B). Consistently, patients with low expression of ATG7 in the breast stroma had shorter overall survival (OS) and relapse-free survival (RFS), whereas varying ATG7 expression levels in the breast epithelium showed no significant correlation with OS and RFS (Fig. 1C). Employing Kaplan‒Meier plotter online web tool and XENA online tools to analyze ATG7 expression in online databases, we affirmed the association of low ATG7 expression with unfavorable prognostic outcomes, including OS, Distant Metastasis-Free Survival (DMFS) and RFS (Supplementary Fig. 1C). Consistently, GEO database (GSE100534) analysis revealed that diminished expression of ATG7 was evident in metastatic breast cancer tissues compared to primary breast tissues (Supplementary Fig. 1D). In summary, our comprehensive investigation reveals a pivotal role for ATG7 in breast cancer progression, with its distinct impact on stroma significantly influencing patient outcomes.

As highlighted earlier, fibroblasts constitute the predominant stromal cell population in breast cancer. Our hypothesis posits that the poor prognosis observed in breast cancer patients is primarily due to the diminished expression of *ATG7* within the fibroblasts. Utilizing microarray data retrieved from the GEO database (GSE27018), we found that compared with that in monoculture cells, *ATG7* expression in breast cancer epithelial cells was retained when cocultured with breast tumor tissue fibroblasts (Fig. 1D). However, further examination of primary fibroblasts derived from breast cancer patients by GEO database analysis of GSE29270 indicated that CAFs showed significantly lower *ATG7* expression than normal fibroblasts (Fig. 1E). Consequently, these findings prompt us to consider that the expression level of ATG7 in fibroblasts might hold greater relevance in influencing the progression of breast cancer.

A large amount of data shows that 25–50% of patients diagnosed with breast cancer will eventually develop fatal metastases, and the presence of residual disease and distant metastasis after initial treatment usually leads to recurrence after several years to decades. In addition, recurrent breast cancer is usually characterized by a high proportion of invasive cells [35–38]. Primary breast cancer cells metastasize to various distant organs through blood vessels, preferentially metastasizing to the lungs, liver and bones [39, 40]. Therefore, to confirm these clinical observations and explore the molecular mechanism of patient relapse, we first examined the effect of *Atg7* expressed in fibroblasts on the progression of breast tumors in an orthotopic mouse model of spontaneous breast cancer metastasis. After subcutaneously injecting 4T1 (BALB/C mouse) or MDA-MB-231 (BALB/C-NU mouse) cells mixed with wild-type (WT) or *Atg7*^−/−^ MEFs into mammary fat pad, the cancer cells mixed with *Atg7*^−/−^ MEFs both generated tumors of greater volume and weight than that mixed with WT MEFs or injected alone, respectively (Fig. 1F, G, Supplementary Fig. 1E–G). Moreover, bioluminescence imaging also showed that tumors in the *Atg7*^−/−^ MEFs group grew larger (Fig. 1H, Supplementary Fig. 1H). In addition, notable organ metastatic colonization was observed in the mice injected with 4T1 or MDA-MB-231 cells mixed with *Atg7*^−/−^ MEFs, as measured by bioluminescence imaging and quantified based on the number of metastases (Fig. 1I, Supplementary Fig. 1I). Further analysis based on the hematoxylin and eosin staining of lung tissue also confirmed these observations (Fig. 1J, Supplementary Fig. 1J). Thus, these results demonstrated that *Atg7*^−/−^ fibroblasts can promote breast cancer proliferation and metastasis compared with WT fibroblasts.

### *Atg7* deletion converts fibroblasts into activated fibroblasts exhibiting the features of CAFs

**Fig. 2:**
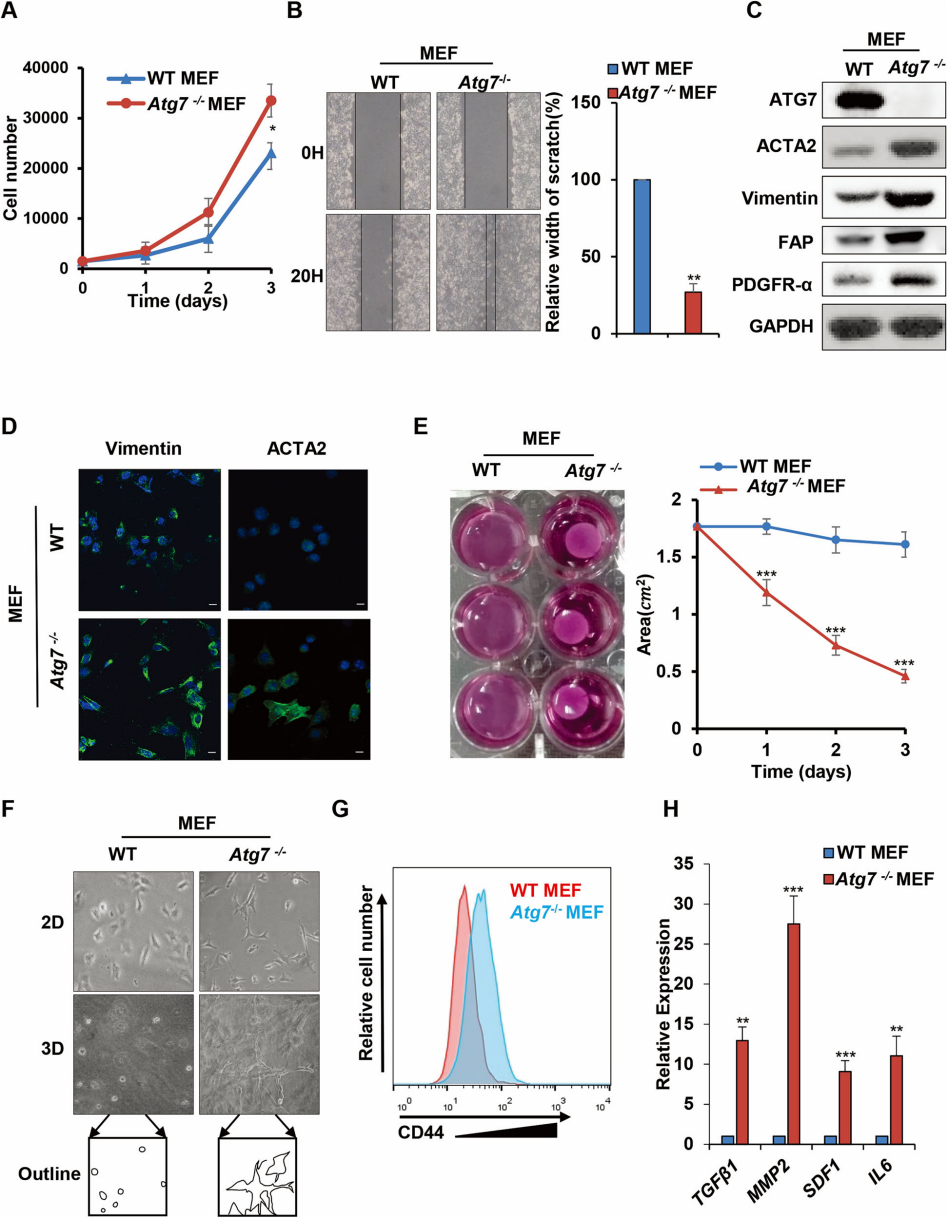
*Atg7* deletion converts fibroblasts into activated fibroblasts exhibiting features of CAFs. **A** The growth numbers of WT MEFs and *Atg7*^−/−^ MEFs were recorded at 1 day, 2 days and 3 days. **B** Scratch assays of WT MEFs and *Atg7*^−/−^ MEFs. **C** Western blot analysis of ATG7, ACTA2, Vimentin, PDGFR-α, FAP protein levels in WT MEFs and *Atg7*^−/−^ MEFs. **D** Immunofluorescence staining of Vimentin and ACTA2 in WT MEFs and *Atg7*^−/−^ MEFs. DAPI-stained nuclei are blue. Scale bar = 15 μm. **E** Collagen contraction assay of WT MEFs and *Atg7*^−/−^ MEFs. Representative images of three replicates of each group at 3 days are shown. **F** The morphology and outline of WT MEFs and *Atg7*^−/−^ MEFs in the collagen lattice are described by 2D and 3D culture. **G**. Flow cytometry analysis of CD44 expression in WT MEFs and *Atg7*^−/−^ MEFs. **H**. Quantitative PCR analysis of TGF-β1, MMP2, SDF-1 and IL-6 expression in WT MEFs and *Atg7*^−/−^ MEFs. Data are presented as mean ± s.d. of three independent experiments and analyzed by Student’s *t* test. NS not significant, *p < 0.05; **p < 0.01; ***p < 0.001.

The above in vivo results encouraged us to explore the molecular regulation mode. The aberrant expression of certain specific genes in fibroblasts has been shown to be one of a key driver of the conversion of normal tissue-resident fibroblasts into active fibroblasts (CAFs-like), which play an active role in remodeling the TME and substantially promote tumorigenesis [41]. We next explored whether *ATG7* deletion stimulates fibroblast activation and thereby promotes breast tumor progression. To investigate the phenotype of *Atg7* deletion by fibroblasts, their activation properties were evaluated. As shown in Fig. 2A, B, *Atg7*^−/−^ MEFs showed somewhat enhanced proliferation capacity and migration ability. Activated fibroblasts are usually characterized by increased expression of FAP, PDGFR, ACTA2, Vimentin and enhanced contractile properties [42]. Immunoblot and immunofluorescence data indicated that *Atg7* deletion in MEFs significantly promoted the expression of FAP, PDGFR, ACTA2 and Vimentin (Fig. 2C,D). Similarly, *Atg7*^−/−^ MEFs clearly enhanced collagen gel contraction compared with WT MEFs (Fig. 2E). Moreover, *Atg7*^−/−^ MEFs exhibited obvious stretched morphology in dishes (two-dimensional culture) and collagen gels (three-dimensional culture) (Fig. 2F). CD44 is another essential marker of the activated fibroblast phenotype in the TME that was recently considered. Consistently, in Fig. 2G, enhanced CD44 expression and accumulation on the surface of *Atg7*^−/−^ MEFs was observed by flow cytometry. Activated fibroblasts can secrete more cytokines such as MMP-2, TGF-β, SDF-1, and IL-6 for extracellular matrix (ECM) remodeling and tumor progression [12, 43]. Here, we found that *Atg7*^−/−^ MEFs expressed increased levels of cytokines, including *TGF-β*, *MMP2*, *SDF-1* and *IL6*, compared with WT MEFs (Fig. 2H). Finally, we performed ATG7 rescue experiments to confirm that the observed phenotypes of *Atg7*^−/−^ MEFs were due to ATG7 deficiency (Supplementary Fig. 2A–D). In summary, these data revealed that *Atg7* crucially regulates the fibroblast phenotype and that *Atg7* deletion promotes fibroblast conversion into activated fibroblasts.

### Fibroblasts with *Atg7* deficiency promote the proliferation, metastasis and stemness of cancerous mammary epithelial cells

**Fig. 3:**
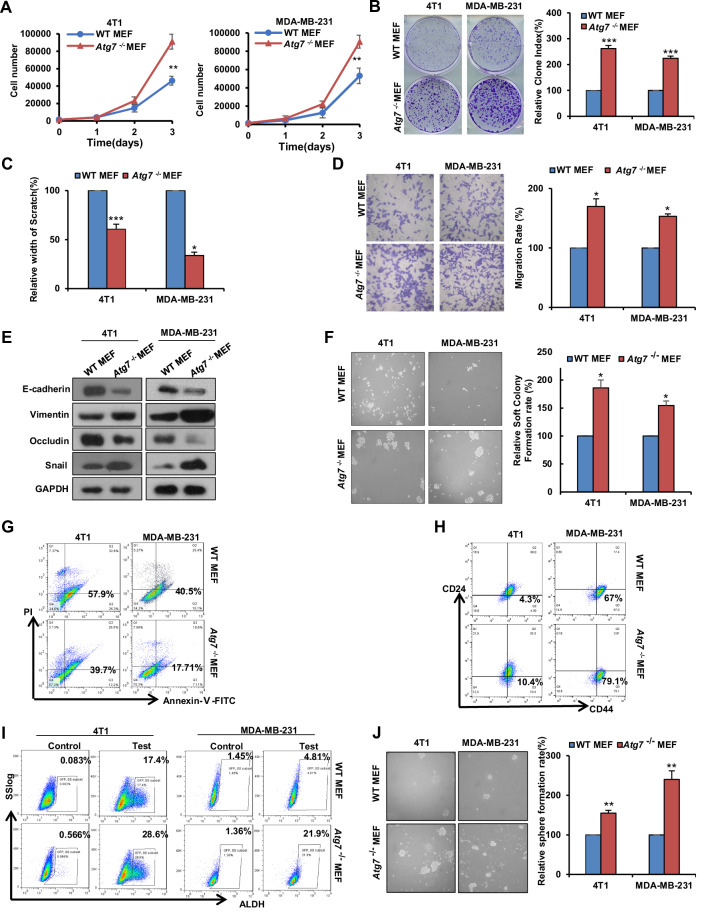
Fibroblasts with *Atg7* deficiency promote the proliferation, metastasis and stemness of cancerous mammary epithelial cells. **A** Growth numbers of 4T1 and MDA-MB-231 cells were recorded after the indicated CM treatment for 1 day, 2 days and 3 days. **B** The viability of 4T1 and MDA-MB-231 cells was assessed by the colony formation assay after the indicated CM treatment. **C** Scratch assays of 4T1 and MDA-MB-231 cells treated with the indicated CM for 20 h. Statistical data are shown. **D** Transwell assays of 4T1 and MDA-MB-231 cells treated with the indicated CM for 20 h. Representative images are shown, and the migrated cells were counted. **E** Western blot analysis of E-cadherin, Vimentin, Occludin and Snail protein levels in the indicated CM-treated 4T1 and MDA-MB-231 cells. GAPDH was used as a loading control. **F** Representative images (left) and quantification (right) of colony formation in soft agar from the indicated CM-treated 4T1 and MDA-MB-231 cells. **G** Flow cytometry analysis of the apoptotic cell population in the indicated CM-treated 4T1 and MDA-MB-231 cells. **H** Flow cytometry analysis of the cancer stem-like cell population (CD44^high^/CD24^low^) in the indicated CM-treated 4T1 and MDA-MB-231 cells. **I** Flow cytometry analysis of the cancer stem-like cell population (ALDH1^high^) in the indicated CM-treated 4T1 and MDA-MB-231 cells. **J** Representative images (left) and quantification (right) of spheroid formation from the indicated CM-treated 4T1 and MDA-MB-231 cells. Data are presented as mean ± s.d. of three independent experiments and analyzed by Student’s *t* test. NS not significant, *p < 0.05; **p < 0.01; ***p < 0.001.

To examine the effect of fibroblasts on breast cancer cells through paracrine crosstalk, we treated 4T1 and MDA-MB-231 cells with prepared conditioned medium (CM) from WT and *Atg7*^−/−^ MEFs and then examined cell proliferation. As shown in Fig. 3A, B, *Atg7*^−/−^ MEFs promoted the proliferation and plate colony formation of 4T1 and MDA-MB-231 cells compared with WT MEFs. Correspondingly, Scratch/Transwell assays also indicated that *Atg7*^−/−^ MEFs exhibited stronger induction of breast cancer epithelial cell migration (Fig. 3C, D, Supplementary Fig. 2E). Similar results were obtained for the breast cancer cell lines T47D and MDA-MB-468 (Supplementary Fig. 2F–I). Epithelial-mesenchymal transition (EMT) is one of the crucial mechanisms that regulates the initial steps of metastatic progression. Further measurement of the expression of EMT markers showed that the mesenchymal markers Vimentin and Snail were upregulated while the epithelial markers E-cadherin and Occludin were downregulated when 4T1 and MDA-MB-231 cells were treated with *Atg7*^−/−^ MEFs CM compared with those treated with WT (Fig. 3E). Multicellular survival and anchorage-independent growth occurred when cells were removed from inappropriate cell/ECM interactions, these abilities are closely related to the malignant and metastatic potential of cells [44]. The Anoikis and soft agar colony formation in Fig. 3F, G show that *Atg7*^−/−^ MEFs CM markedly inhibited anoikis and promoted anchorage-independent growth. The most consistently used biomarkers for identifying breast cancer stem cells (BCSC) are CD44, CD24, and ALDH1 [45]. Evidence suggests that BCSCs with ALDH1 positivity and the CD44high/CD24low phenotype contribute to tumor initiation, progression, metastasis, and drug resistance [46]. Moreover, Spheroid formation assays are used to evaluate the self-renewal and tumor-initiating potential of BCSCs. Here, flow cytometry analysis of CD44high/CD24low cells indicated that *Atg7*^−/−^ MEFs also promoted the stemness of 4T1 and MDA-MB-231 cells (Fig. 3H). Further ALDH expression analysis and spheroid formation cell assays also confirmed this result (Fig. 3I, J). Interestingly, when ATG7 was restored in *Atg7*^−/−^ MEFs, its promoting effect on breast cancer cells was significantly reduced (Supplementary Fig. 2J–M). Therefore, these results indicate that compared with WT fibroblasts, *Atg7*-deficient fibroblasts promote the proliferation, metastasis and stemness of breast cancer cells through paracrine signals.

### Exosomes from *Atg7*-deficient fibroblasts contribute to breast cancer-promoting effects

**Fig. 4:**
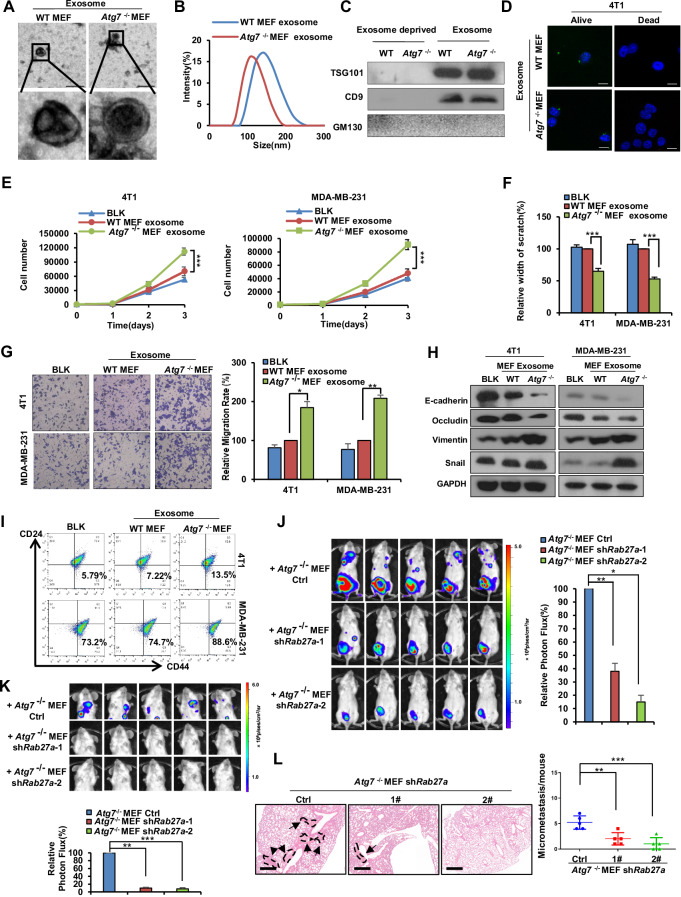
Exosomes from *Atg7*-deficient fibroblasts contribute to breast cancer-promoting effects. **A** Exosomes isolated from indicated MEFs were detected by electron microscopy. Scale bar, 200 nm. **B** Nanoparticle tracking analysis of exosomes isolated from indicated MEFs. **C** Western blot analysis of TSG101, CD9 and GM130 in exosome-deprived CM and exosomes from indicated MEFs. **D** Confocal imaging of DAPI-labeled 4T1 cells incubated with DiD-labeled exosomes (green) for 24 h. Scale bar, 15 μm. **E** Growth numbers of 4T1 and MDA-MB-231 cells were recorded after treatment with the indicated exosomes for 1 day, 2 days and 3 days. **F** Scratch assays of 4T1 and MDA-MB-231 cells treated with the indicated exosomes for 20 h. Statistical data are shown. **G** Transwell assays of 4T1 and MDA-MB-231 cells treated with the indicated exosomes for 20 h. Representative images are shown, migrated cells were counted. **H** Western blot analysis of E-cadherin, Vimentin, Occludin and Snail protein levels in the indicated exosome-treated 4T1 and MDA-MB-231 cells. **I** Flow cytometry analysis of the cancer stem-like cell population (CD44^high^/CD24^low^) in the indicated exosome-treated 4T1 and MDA-MB-231 cells. **J**. Xenograft tumors of 4T1-Luc cells mixed with indicated MEFs were quantified using bioluminescence imaging. Representative in vivo bioluminescent images are shown. **K** Metastatic colonization of 4T1-Luc cells was quantified using bioluminescence imaging. Representative in vivo bioluminescent images are shown. **L** Left: Representative H&E-stained sections of lungs 4T1-Luc xenograft mice, metastases are indicated by the arrows. Right: Quantification of metastatic colonization in the lungs. Data are presented as mean ± s.d. of three independent experiments and analyzed by Student’s *t* test. NS not significant, *p < 0.05; **p < 0.01; ***p < 0.001.

Crosstalk between tumor cells and the TME is mainly mediated by direct cell‒cell contacts, secretory soluble small molecules and extracellular vesicles (EVs), which are classified as microvesicles (100–1000 nm), exosomes (30–200 nm), or apoptotic bodies (generally > 1000 nm) depending on their size, biogenesis mechanisms, or function [47]. Among them, exosomes are EVs released by all cell types and play an important role in mediating the exchange of information between cancer and stromal cells within the TME [48]. To determine whether free secreted factors or exosomes in the supernatant play a role in promoting breast cancer progression, we obtained exosome- and exosome-deprived supernatant by ultracentrifugation from fibroblasts. Electron microscopy observations and a particle size analysis of isolated exosomes indicated that MEFs can secrete exosomes and our isolated exosomes were of high quality (Fig. 4A, B). Western blot analysis of the exosome markers TSG101 and CD9 and the Golgi membrane protein GM130 further verified that we successfully isolated exosomes without cellular contamination (Fig. 4C). Through bicinchoninic acid protein analysis, we also found that WT MEFs secreted almost the same number of exosomes as *Atg7*^−/−^ MEFs (Supplementary Figure 2N). To identify exosome internalization by cancer cells, purified exosomes were labeled with the fluorescent lipophilic dye DiD and incubated with 4T1 cells. The fluorescence microscopy results showed the presence of DiD spots in living recipient 4T1 cells but not dead (fixed) cells, which indicated that the exosomes collected from various MEFs were transferred into living cancer cells via active transport (Fig. 4D).

To evaluate whether exosomes or exosome-deprived supernatant is sufficient to induce protrusive activity in breast cancer cells, we treated MDA-MB-231 and 4T1 cells with different isolated exosomes or exosome-deprived supernatant. Interestingly, we found that exosomes derived from *Atg7*^−/−^ MEFs notably promoted MDA-MB-231 and 4T1 cells proliferation, metastasis, EMT and stemness (Fig. 4E–I, Supplementary Fig. 3A). However, exosome-deprived supernatant did not significantly promote breast cancer function (Supplementary Fig. 3B–F). Therefore, these results preliminarily suggest that exosomes, rather than exosome-deprived supernatant from fibroblasts, may promote the progression of breast cancer.

To further verify the function of exosomes, *Atg7*^−/−^ MEFs were treated with GW4869, an N-Smase inhibitor that blocks exosome generation, and the CM was then collected for subsequent functional studies. Inhibiting the production of exosomes caused the CM to lose the ability to promote cell proliferation, metastasis, EMT and stemness (Supplementary Fig. 3G–K). RAB27A, a small GTPase, is critically involved in vesicle transport and regulates the release of exosomes [49]. To further investigate the role of exosomes in the crosstalk between fibroblasts and breast cancer cells, we generated *Atg7*^−/−^ MEFs in which *Rab27a* was constitutively knocked down by short hairpin RNAs (shRNAs), resulting in cells with abnormal exosomal function (Supplementary Fig. 4A,B). The results showed that *Rab27a* knockdown significantly reduced the ability of *Atg7*^−/−^ MEFs to stimulate breast cancer cell malignant progression (Supplementary Fig. 4C–G). Moreover, similar results were observed in vivo (Fig. 4J–L, Supplementary Fig. 4H, I).

Taken together, these results reveal that *Atg7*^−/−^ fibroblasts promote breast cancer cell proliferation, induce EMT and increase tumor metastasis through paracrine signals of exosomes.

### miR-6803b in exosomes secreted by *ATG7*^−/−^ fibroblasts plays a crucial role in promoting breast cancer progression

**Fig. 5:**
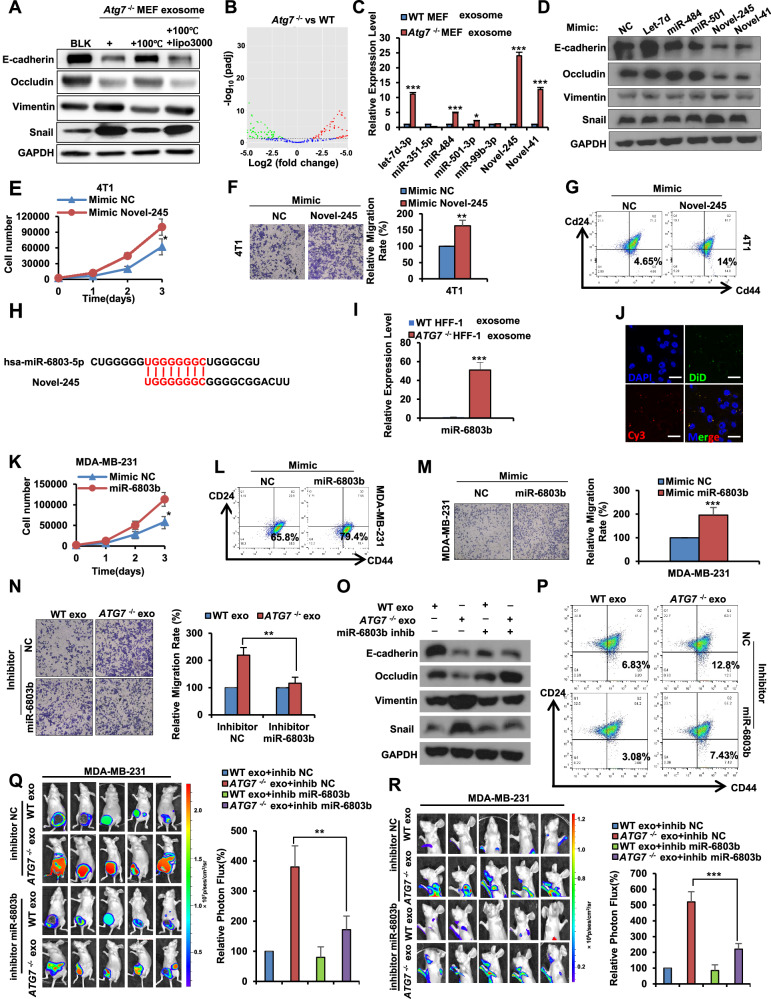
miR-6803b in exosomes secreted by *Atg7*^−/−^ fibroblasts plays a crucial role in promoting breast cancer progression. **A** Western blot analysis of E-cadherin, Vimentin, Occludin and Snail levels in the indicated exosomes (exosomes of *Atg7*^*−/−*^ MEFs were treated with a temperature of 100 °C) treated 4T1 cells. **B** Small RNAseq of exosome is presented in volcano plot. **C** QPCR analysis of the top 7 miRNAs in exosomes. **D** Western blot analysis of E-cadherin, Vimentin, Occludin and Snail levels in indicated miRNA mimic treated 4T1 cells. **E** Growth numbers of 4T1 cells were recorded after the indicated miRNA mimic or control treatment. **F** Transwell assays of 4T1 cells treated with indicated miRNA mimic or control. **G** Flow cytometry analysis of the cancer stem-like cell population (CD44high/CD24low) in 4T1 cells. **H**. Mature body sequences and paired seed sequences of Novel-245 and hsa-miR-6803-5p. **I**. QPCR analysis of the miR-6803b in exosomes from indicated HFF-1 cells. **J** Confocal imaging showed the delivery of Cy3-labeled miR-6803b (red) and DiD-labeled exosomes (green) to DAPI-labeled MDA-MB-231 cells (blue). Scale bar, 15 μm. **K** Growth numbers of MDA-MB-231 cells were recorded after indicated miRNA mimic treatment. **L** Flow cytometry analysis of the cancer stem-like cell population (CD44high/CD24low) in MDA-MB-231 cells. **M** Transwell assays of MDA-MB-231 cells treated with indicated miRNA mimic. **N** Transwell assays of MDA-MB-231 cells treated with indicated exosomes or miRNA inhibitor. **O** Western blot analysis of E-cadherin, Vimentin, Occludin and Snail protein levels in indicated exosomes or miRNA inhibitor-treated MDA-MB-231 cells. **P** Flow cytometry analysis of the cancer stem-like cell population (CD44high/CD24low) in indicated exosomes or miRNA inhibitor-treated MDA-MB-231 cells. **Q** Xenograft assays of MDA-MB-231-Luc cells. *ATG7*^*−/−*^ HFF-1 exosomes or miR-6803b inhibitor are injected into tumors. **R**. Metastatic colonization of MDA-MB-231-Luc cells was quantified using bioluminescence imaging. Data are presented as mean ± s.d. of three independent experiments and analyzed by Student’s *t* test. NS not significant, *p < 0.05; **p < 0.01; ***p < 0.001.

The selectively packaged nucleic acid (RNA) and protein in exosomes are the two main components that mediate intercellular communication [47]. In our pursuit to identify factors within exosomes that drive breast cancer progression, purified exosomes were subjected to heating (100 °C, 15 min), followed by treatment of 4T1 cells alone or in conjunction with Lipo3000. The results revealed that heating eliminates the ability of exosomes to promote cell EMT, but liposomes can partially rescue the activity of exosomes, which implied that the active factor may be RNA rather than protein (Fig. 5A). To pinpoint the specific RNA involved, we conducted microarrays to generate small RNA seq of exosomes derived from WT and *Atg7*^−/−^ MEFs, and the results are shown as heatmaps and volcano plots in Fig. 5B and Supplementary Fig. 5A. Subsequently, we identified the top 7 significantly differentially expressed miRNAs, with detailed sequencing information provided in Supplementary Fig. 5B. QPCR analysis of exosomal miRNA unveiled heightened expression of let-7d-3p, miR-484, miR-501-3p, Novel-245, and Novel-41 in Atg7^−/−^ MEFs compared to WT MEFs, remarkably, the expression level of Novel-245 showed the most significant difference (Fig. 5C). Treating 4T1 cells with these 5 types of miRNA mimics showed that Novel-245, a new kind of miRNA, can significantly induce EMT in cancer cells (Fig. 5D). Subsequently, a series of in vitro experiments demonstrated that Novel-245 actively promotes proliferation, metastasis, and stemness of breast cancer cells (Fig. 5E–G, Supplementary Fig. 5C). To prove that the new miRNA we discovered, Novel-245, also exists in *Homo sapiens*, we firstly confirmed that its host gene is located on Homo sapiens chromosome 9 by BLASTing its precursor sequence (Supplementary Fig. 5D). Next, using the mature Novel-245 sequence, we predicted the seed sequence as UGGGGGGC in the miRDB database, a parallel approach was applied to BLAST human miRNAs with identical seed sequences, hsa-miR-6803-5p, on the miRcarta database (Fig. 5H). Therefore, in adherence to miRbase naming rules for new miRNAs, we tentatively designated this novel miRNA as miR-6803b. Here, we also identified high expression of miR-6803b in exosomes from human fibroblasts HFF-1 *ATG7*^−/−^ cells (Fig. 5I). Besides, miR-6803b in *ATG7*^−/−^ HFF-1 cells can indeed be packaged into exosomes and absorbed by recipient human breast cancer cells—MDA-MB-231 (Fig. 5J). More importantly, miR-6803b mimics can also significantly promote the proliferation, metastasis and stemness of MDA-MB-231 cells (Fig. 5K-M, Supplementary Fig. 5E). Collectively, these findings underscore miR-6803b as a newly discovered functional miRNA exhibiting conservation between humans and mice.

To confirm the role of exosomal miR-6803b, we exposed breast cancer cells to exosomes derived from *ATG7*^−/−^ HFF-1, either in isolation or in combination with a miRNA inhibitor. Our findings, depicted in Fig. 5N–P and Supplementary Fig. 5F, G, reveal that the miRNA inhibitor effectively attenuated the positive impact of exosomes on breast cancer cell progression, encompassing facets such as cell proliferation, metastasis, EMT, and stemness. Similar results were observed in vivo (Fig. 5Q, R, Supplementary Fig. 5H). Thus, our results suggest that miR-6803b in exosomes secreted by *ATG7*^−/−^ fibroblasts, as a messenger from fibroblasts to cancer cells, plays a crucial role in inducing breast cancer progression and promoting tumorigenesis.

### Exosomal miR-6803b promotes breast cancer progression by targeting *SCARB1*

**Fig. 6:**
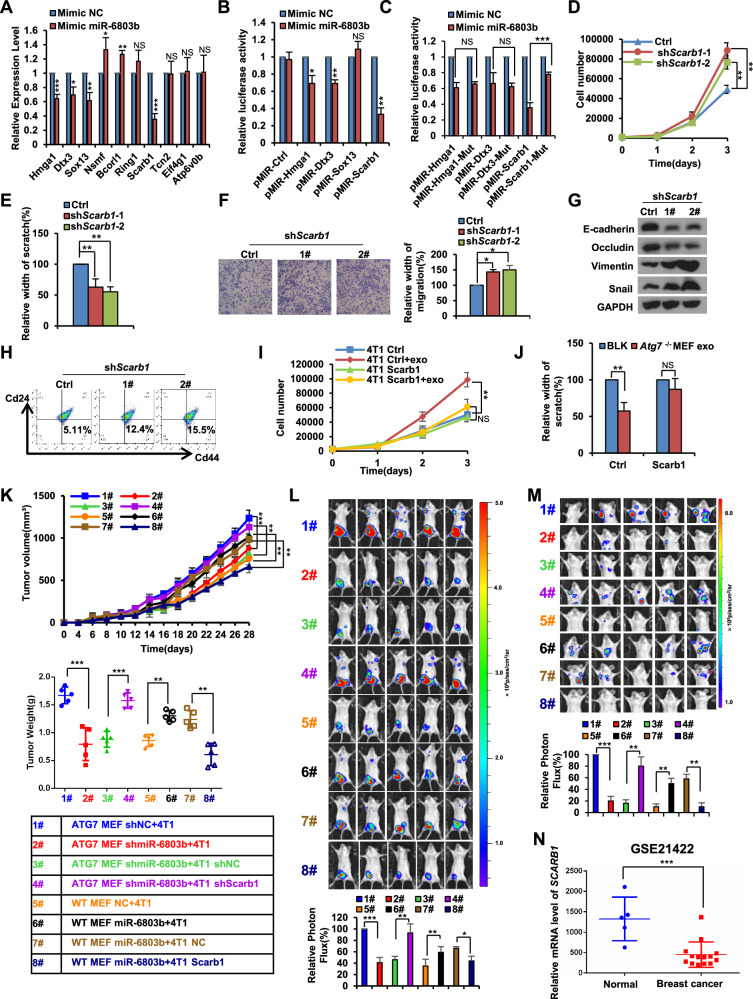
Exosomal miR-6803b promotes breast cancer progression by targeting *SCARB1.* **A** QPCR analysis of the top 10 target genes in 4T1 cells treated with miR-6803b mimic. **B**, **C** Relative luciferase activity of 4T1 cells in the presence of the indicated treatments. **D** Growth numbers of shScarb1-expressing 4T1 cells and control cells were recorded at 1 day, 2 days and 3 days. **E** Scratch assays of shScarb1-expressing 4T1 cells and control cells. Statistical data are shown. **F** Transwell assays of shScarb1-expressing 4T1 cells and control cells. **G** Western blot analysis of E-cadherin, Vimentin, Occludin and Snail protein levels in shScarb1-expressing 4T1 cells and control cells. **H** Flow cytometry analysis of the cancer stem-like cell population (CD44^high^/CD24^low^) in shScarb1-expressing 4T1 cells and control cells. **I** The growth numbers of Scarb1-overexpressing 4T1 cells and control cells were recorded after treatment with the indicated exosomes. **J** Scratch assays of shScarb1-overexpressing 4T1 cells and control cells treated with the indicated exosome. Statistical data are shown. **K** Xenograft assays of 4T1-Luc cells (infected with lentiviruses carrying Scarb1 overexpression or shScarb1) mixed with WT MEFs (infected with lentiviruses carrying miR-6803b overexpression) or *Atg7*^*−/−*^ MEFs (infected with lentiviruses carrying shmiR-6803b). Tumor growth curves (above) and scatterplots of the individual weights of tumors (middle) are shown. The groups represented by the numbers are listed in the table (below). **L** Representative in vivo bioluminescent images are shown. **M** Representative in vivo bioluminescent images of metastatic colonization are shown. **N**. *SCARB1* expression levels in normal tissues and breast cancer from the GEO database (GSE21422). Data are presented as mean ± s.d. of three independent experiments and analyzed by Student’s *t* test. NS not significant, *p < 0.05; **p < 0.01; ***p < 0.001.

To determine the targets of exosomal miR-6803b in breast cancer cells, a bioinformatics tool miRDB were used to predict the target genes based on mature miR-6803b sequences. After intersecting with the predicted targets in miRNA sequencing and further screening, we found 10 potential genes that may be its target genes of miR-6803b (Supplementary Fig. 6A, B). To determine the function of these genes, we treated cells with the miR-6803b mimic and then assayed the mRNA levels of these 10 genes by QPCR. The results showed that the miR-6803b mimic significantly reduced the RNA levels of *Hmga1*, *Dtx3*, *Sox13* and *Scarb1* (Fig. 6A). Then, the binding sites of miR-6803b in wild-type *Hmga1*, *Dtx3*, *Sox13* and *Scarb1* were cloned into luciferase vectors and cotransfected into 4T1 cells with miR-6803b mimic. Luciferase activity decreased markedly in 4T1 cells cotransfected with the *Hmga1*, *Dtx3* and Scarb1 wild-type binding site vectors in the presence of miR-6803b (Fig. 6B). However, when cells contained the *Scarb1* mutated binding site vector, luciferase activity did not show such repression when cotransfected with the miR-6803b mimic (Fig. 6C). Thus, these results indicate that Scarb1 is probably a direct target of miR-6803b in breast cancer cells.

Next, a set of experiments in vitro and in vivo were conducted to further confirm that miR-6803b targets Scarb1 to promote breast cancer progression. First, the knockdown of Scarb1 imitated the promotion of miR-6803b on breast cancer cell proliferation, migration and invasion, EMT development and stemness (Fig. 6D–H and Supplementary Fig. 6C, D). Consistently, the overexpression of Scarb1 also inhibited the role of exosomes in promoting the proliferation and migration of breast cancer cells (Fig. 6I, J, and Supplementary Fig. 6E). Notably, we also found that miR-6803b promotes breast cancer proliferation and metastasis by targeting Scarb1 in vivo (Fig. 6K–M and Supplementary Fig. 6F, G). Finally, using GEO database (GSE21422) and Kaplan‒Meier plotter online web tool revealed that *SCARB1* expression was significantly decreased in breast cancer (Fig. 6N), and a high expression of *SCARB1* was significantly associated with worse OS, RFS and DMFS for BRCA patients (Supplementary Fig. 6H). Therefore, the above results suggest that exosomal miR-6803b derived from fibroblasts promotes breast cancer progression by targeting SCARB1.

### Effects of fibroblasts derived from breast cancer patients on breast cancer cells

**Fig. 7:**
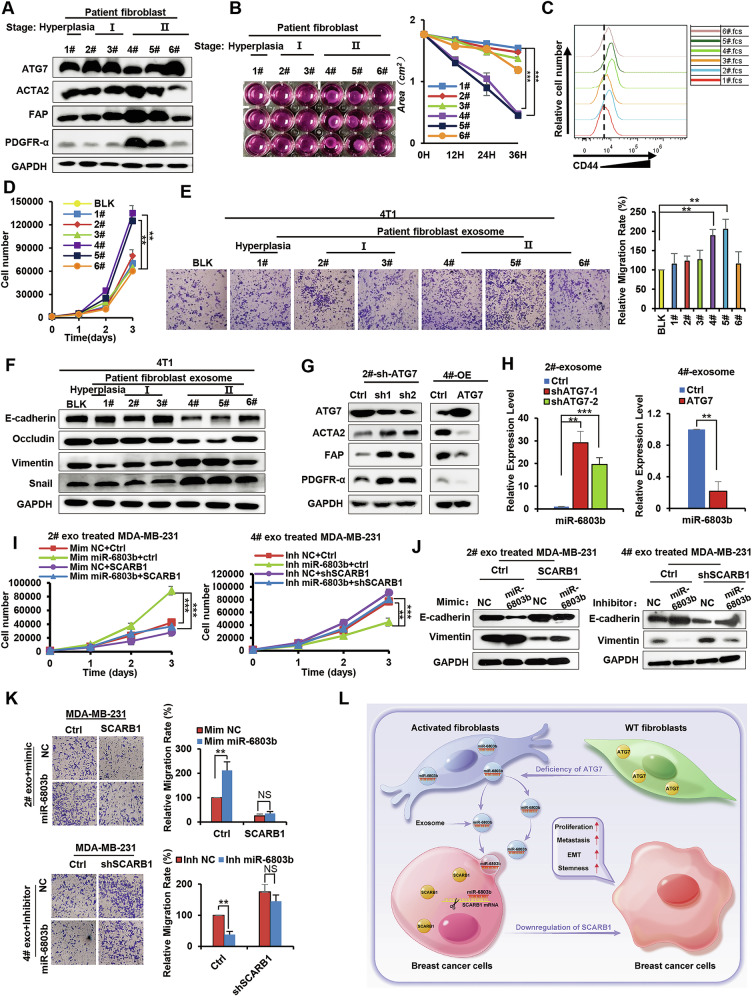
Effects of fibroblasts derived from breast cancer patients on cancer cells. **A** Western blot analysis of ATG7, ACTA2, FAP, and PDGFR protein levels in fibroblasts from breast cancer patient samples. Hyperplasia indicates patients with hyperplasia of the breast, I indicates patients with TNM stage I breast cancer, II indicates patients with TNM stage II breast cancer. **B** Collagen contraction assay of fibroblasts from patient samples. **C** Flow cytometry analysis of CD44 expression in patient fibroblasts. **D** Growth numbers of 4T1 cells were recorded after treatment with the indicated exosomes. **E** Transwell assays of 4T1 cells treated with the indicated exosomes. Representative images are shown, and the migrated cells were counted. **F** Western blot analysis of E-cadherin, Vimentin, Occludin, and Snail protein levels in the indicated exosomes treated with 4T1 cells. **G** Western blot analysis of ATG7, ACTA2, Vimentin, PDGFR-α and FAP protein levels in immortalized patient fibroblasts with ATG7 overexpression (2#) or knockdown (4#). **H** QPCR analysis of miR-6803b in exosome from immortalized patient fibroblasts with ATG7 overexpression (2#) or knockdown (4#). **I** Growth numbers of MDA-MB-231 cells (with Scarb1 overexpression or knockdown) were recorded after treatment with the indicated exosomes (immortalized patient fibroblasts 2# and 4#), miR-6803b mimic or miR-6803b inhibitor. **J** Western blot analysis of E-cadherin and Vimentin protein levels in MDA-MB-231 cells (with Scarb1 overexpression or knockdown) that treated with the indicated exosomes (immortalized patient fibroblasts 2# and 4#), miR-6803b mimic or miR-6803b inhibitor. **K** Transwell assays of MDA-MB-231 cells (with Scarb1 overexpression or knockdown) with the indicated exosomes (immortalized patient fibroblasts 2# and 4#), miR-6803b mimic or miR-6803b inhibitor. **L** A proposed working model. Data are presented as mean ± s.d. of three independent experiments and analyzed by Student’s *t* test. NS not significant, *p < 0.05; **p < 0.01; ***p < 0.001.

To follow up on our findings that *Atg7*-deficient fibroblasts regulate breast cancer progression through paracrine signals in vivo and in vitro, we next investigated the expression and function of *ATG7* in fibroblasts of different breast cancer patient samples. First, we obtained 6 patient samples, including 1 benign breast hyperplasia sample (1#) and 5 breast cancers (samples 2#, 3#, 4#, 5#, 6#), and then isolated and cultured fibroblasts from these tissue samples. The clinicopathological features of the 6 patients are displayed in Supplementary Fig. 7A. Subsequent Western blot analysis showed that fibroblasts derived from patient samples #4 and #5 (TNM stage II) had significantly lower levels of ATG7 protein than fibroblasts from other samples (including 1# hyperplasia and 2# and 3# breast cancer of TNM stage I) (Fig. 7A). Consistent with our previous results (Fig. 3), patient-derived fibroblasts with low expression of ATG7 exhibited activated fibroblast features, including increased gel contraction and migration and enhanced CD44 expression (Fig. 7B, C and Supplementary Fig. 7B). More interestingly, further investigation indicated that exosomes from TNM stage II patients’ fibroblasts (samples #4 and #5) showed an enhanced ability to induce breast cancer 4T1 cell proliferation, migration, EMT and cell stemness (Fig. 7D–F and Supplementary Fig. 7C, D), which is consistent with the results of previous cell and animal experiments. To demonstrate the relevance of our newly described *ATG7*-miR-6803b-*SACRB1* axis to human CAFs, we constructed immortalized patient fibroblasts (samples 2# and 4#) and stably knocked down or overexpressed ATG7 in 2# and 4#, respectively. Western blot results indicated that the expression level of ATG7 is closely related to the activation of human fibroblasts (Fig. 7G). What’s more, QPCR of exosomes from these two groups fibroblasts demonstrated that the expression of ATG7 in human fibroblasts reduced the level of miR-6803b in exosomes (Fig. 7H). Finally, consistent with the results in mouse cells and in vivo, human fibroblast derived exosomal miR-6803b promoted the malignancy of human breast cells by targeting *SCARB1*. (Fig. 7I–K).

Therefore, these results suggest that fibroblasts derived from advanced breast cancer patients showed a tendency to express lower levels of ATG7 protein and can promote breast cancer progression through *ATG7*-miR-6803b-*SACRB1* axis.

## Discussion

TME, a dynamic system orchestrated by intercellular crosstalk between the neoplastic stroma and the epithelium, has been established to be responsible for tumor progression and metastasis, yet the master regulators are largely unknown. In this report, starting from clinical evidence and focusing on the role of *ATG7* expression in the fibroblasts in the progression of breast cancer, we reveal a communication mode between breast cancer cells and fibroblasts that contributes to the generation of the tumor and its progression. In the present study, we demonstrated that abnormally low expression of *ATG7* in the fibroblasts is closely associated with breast tumorigenesis, mainly due to the reprogramming of the expression profile of fibroblasts by *ATG7* as a regulatory gene. Abnormally low levels of *ATG7* promotes the expression of miR-6803b miRNA in fibroblasts and then uses exosomes as a cargo delivery system to transport miR-6803b miRNA to cancer cells, which targets the *SCARB1* gene via paracrine signal to regulate breast cancer proliferation, metastasis, and stemness. Thus, in the present study, we demonstrate that *ATG7* is an important determinant in the regulation of breast cancer progression through cellular crosstalk between cancer cells and their environmental stromal components.

Although *ATG7* plays a crucial role in autophagy by driving the cardinal stages of classical degradative autophagy through ATG8 lipidation, however, the role of autophagy cannot be assessed by depleting a single ATG protein [50]. Increasing data suggest that cells can still perform autophagy even without ATG7, through an alternative pathway that is independent of ATG5 and ATG7, and in this alternative pathway, autophagosomes are generated by the fusion of phagophores with vesicles from the trans-Golgi and late endosomes [51]. Therefore, we cannot judge whether cell autophagy is inhibited by knocking out ATG7. Besides, ATG proteins have extensive biological importance beyond autophagic function [52, 53]. The diversity of the nonautophagy-related functions of *ATG7* endows it with an important role in tumorigenesis and progression. The implication of *ATG7* in cancer is further complicated by its autophagy-independent ability to modulate cell cycle arrest and apoptosis and modulate the efficacy of anticancer treatments [54]. *ATG7* has been indicated to inhibit the expression of the proapoptotic genes *Noxa*, *Puma* and *Bax* by interacting with the p53 protein. Additionally, a loss of *ATG7* could influence cell cycle and then promote proliferation by diminishing p21 expression during metabolic stress [55, 56]. *ATG7* overexpression stabilizes CD44 by upregulating USP28 protein, which in turn modulates stem cell-like properties, invasion and human lung metastasis in bladder cancer [57]. *ATG7* has also been shown to inhibit the Warburg effect by suppressing PKM2 phosphorylation, which reduced the epithelial-mesenchymal transition of tumor cells [58]. These reports are consistent with our findings, which show that *ATG7* may play a key role in the regulation of gene expression and epigenetics. In the present study, our findings suggest that *ATG7* is a pivotal regulator of the expression of miR-6803b, which is an important factor regulating breast cancer progression. However, the mechanisms by which *ATG7* regulates miR-6803b miRNA expression are unclear and remain to be explored in the future.

Notably, although the ATG7 protein showed extensive nonautophagy-related effects, the role of *ATG7* as an autophagy-regulatory gene in reshaping epithelial-stromal interactions to affect tumor progression should also be carefully considered. Autophagy contributes to maintaining intercellular homeostasis as a temporary survival mechanism, which makes it a double-edged sword for tumorigenesis and anticancer therapy [59, 60]. The autophagy-related functions of *ATG7* involve membrane trafficking events that depend on LC3 lipidation [61]. In our study, we found that fibroblasts lacking *ATG7* expression were insensitive to nutrient deprivation-induced autophagy (data not shown). In the TME, numerous drivers of autophagy are induced due to metabolic abnormalities, such as tissue hypoxia, nutrient deficiency, and lactate accumulation [33]. Most current studies suggest that enhanced autophagy may be a tumor suppressor mechanism and that autophagy deficiency can promote the occurrence of cancer [62]. Thus, from this perspective, abnormally low *ATG7* expression in the stromal fibroblasts of breast cancer tissue should disturb the autophagy balance of tumor tissue, which would also facilitate tumor progression and challenge cancer treatment by remodeling the TME. In this study, we observed that patients with low *ATG* expression showed poor clinical outcomes, which is consistent with the hypothesis of possible consequences of low *ATG7* expression. However, the role of *ATG7* in regulating autophagy in breast cancer progression requires further elucidation.

In addition, we tried to explore the molecular mechanism that leads to low expression of ATG7 in fibroblasts. Since non-cancerous cells and cancer cells in the TME interact dynamically, and previous publications also demonstrated that breast cancer cell–educated fibroblasts possess the characteristics of CAFs [63, 64]. Therefore, it is reasonable to believe that cancer cells may also have a regulatory effect on the expression of ATG7 in fibroblasts. Interestingly, through clinical data mining and preliminary in vitro experiments, we found that breast cancer cells significantly induced low expression of ATG7 in fibroblasts compared with normal breast epithelial cells (data not shown). We will further explore the molecular mechanism of this phenotype in future studies. Anyway, combined with the results of this study, our findings more truly reflect the vicious feedback loop in the progression of breast cancer.

## Conclusion

In summary, our results demonstrate that a loss of *ATG7* in fibroblasts can promote the expression and secretion of miR-6803b-enriched exosomes into the TME. Exosomal miR-6803 internalized into cancer cells promotes proliferation, metastasis and stemness by targeting its specific *SCARB1* gene in breast cancer (Fig. 7L). Therefore, exosomal miR-6803b secreted by fibroblasts could be used as an effective prevention and treatment strategy for breast cancer. *ATG7* may also serve as a tumor suppressor in the TME and thus become a new candidate for diagnosis and prognosis.

## Supplementary information

Supplementary Figure

Supplementary Figure Legends

Supplementary Table 1

Supplementary Materials and Methods

Full and uncropped western blots

## Data Availability

Data will be made available, upon reasonable request, by the corresponding author.
