# Supplementary material for: ATG7-deficient fibroblast promotes breast cancer progression via exosome-mediated downregulation of SCARB1
Source: Cell Death Dis. 2025 Jul 24;16(1):556. doi: 10.1038/s41419-025-07885-6 (PMC12289893; doi:10.1038/s41419-025-07885-6)
Supplement: Supplementary file 1 — Supplementary Figure [file 41419_2025_7885_MOESM1_ESM.pdf]

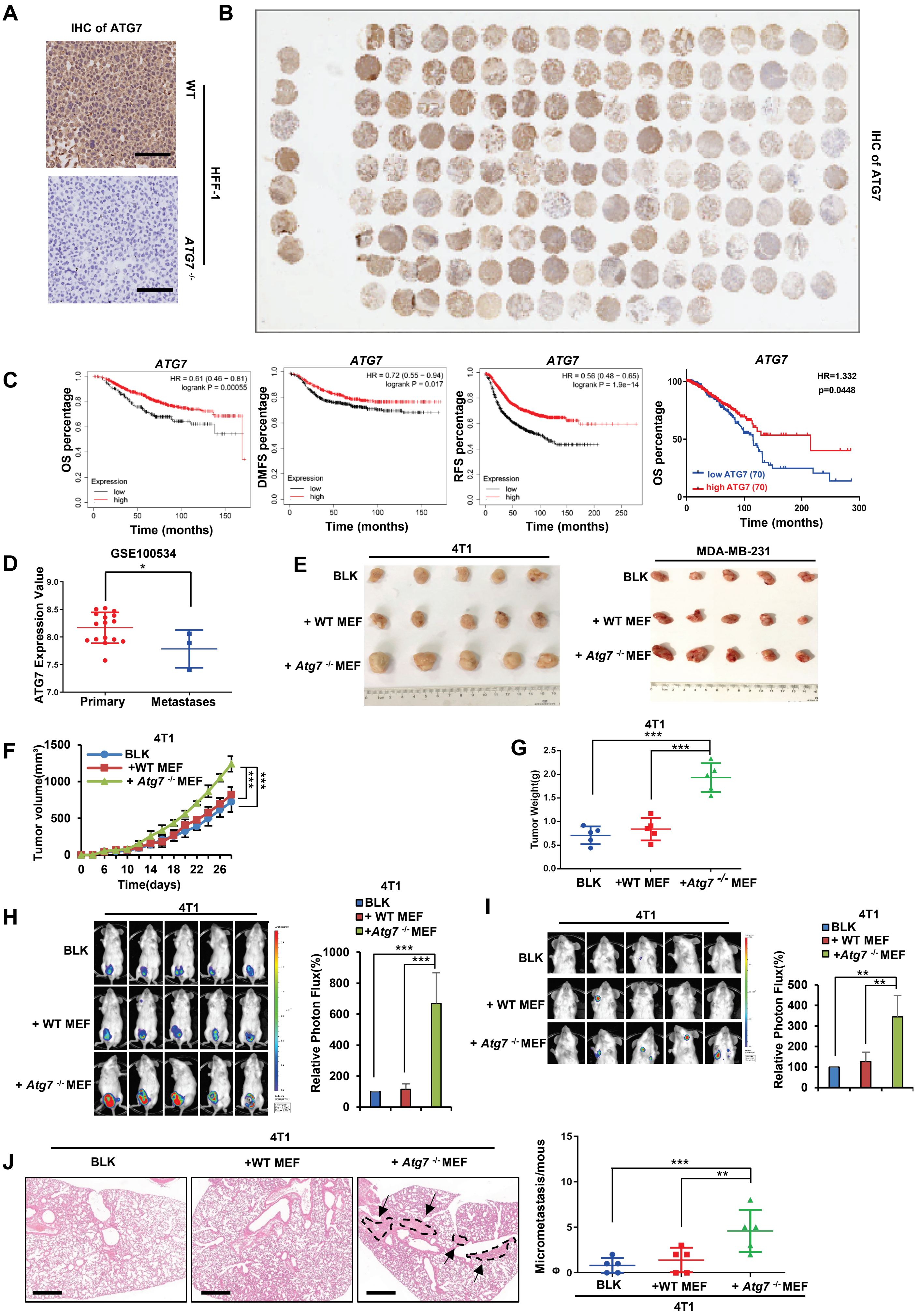

Supplementary Figure 1

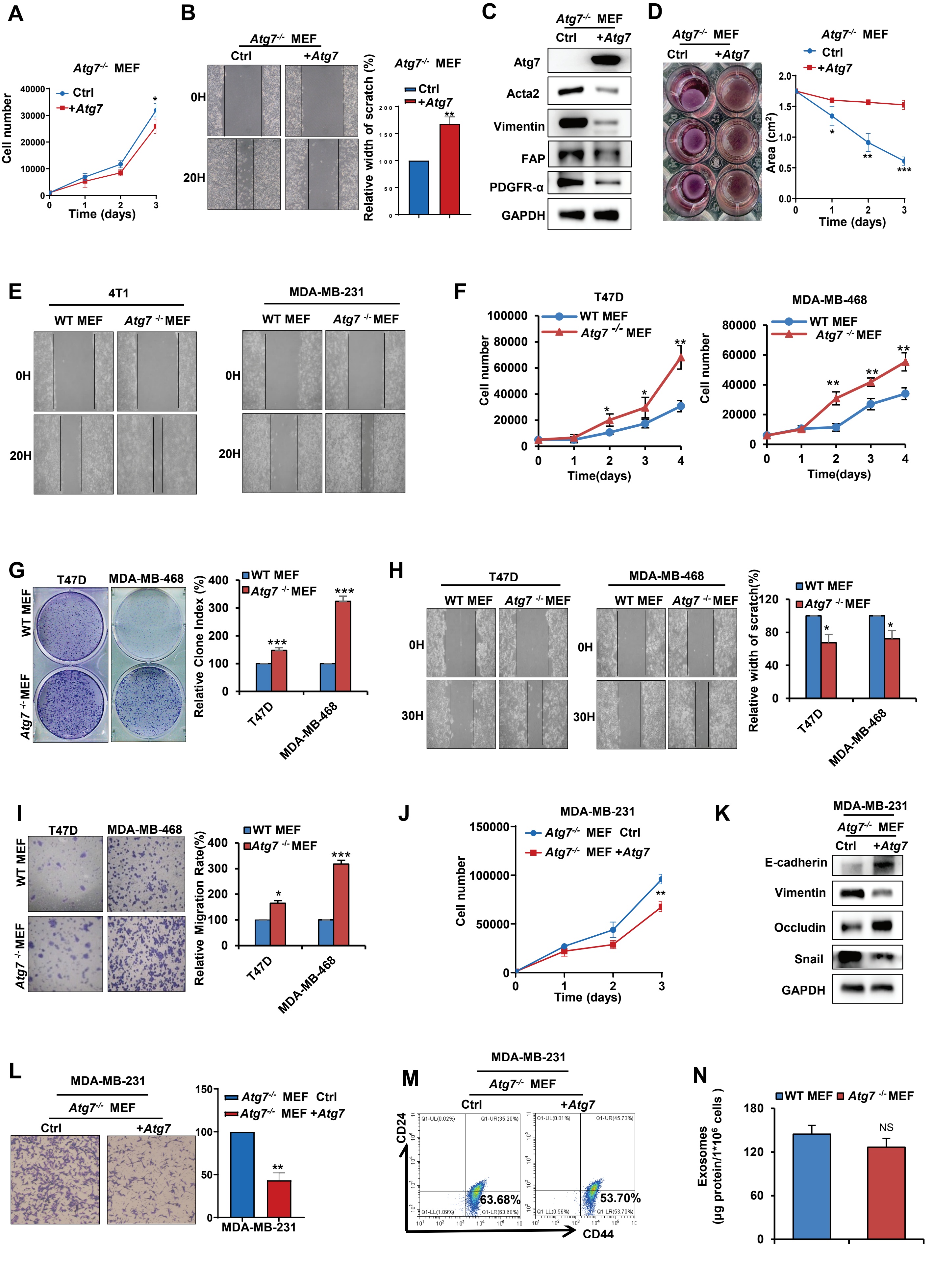

Supplementary Figure 2

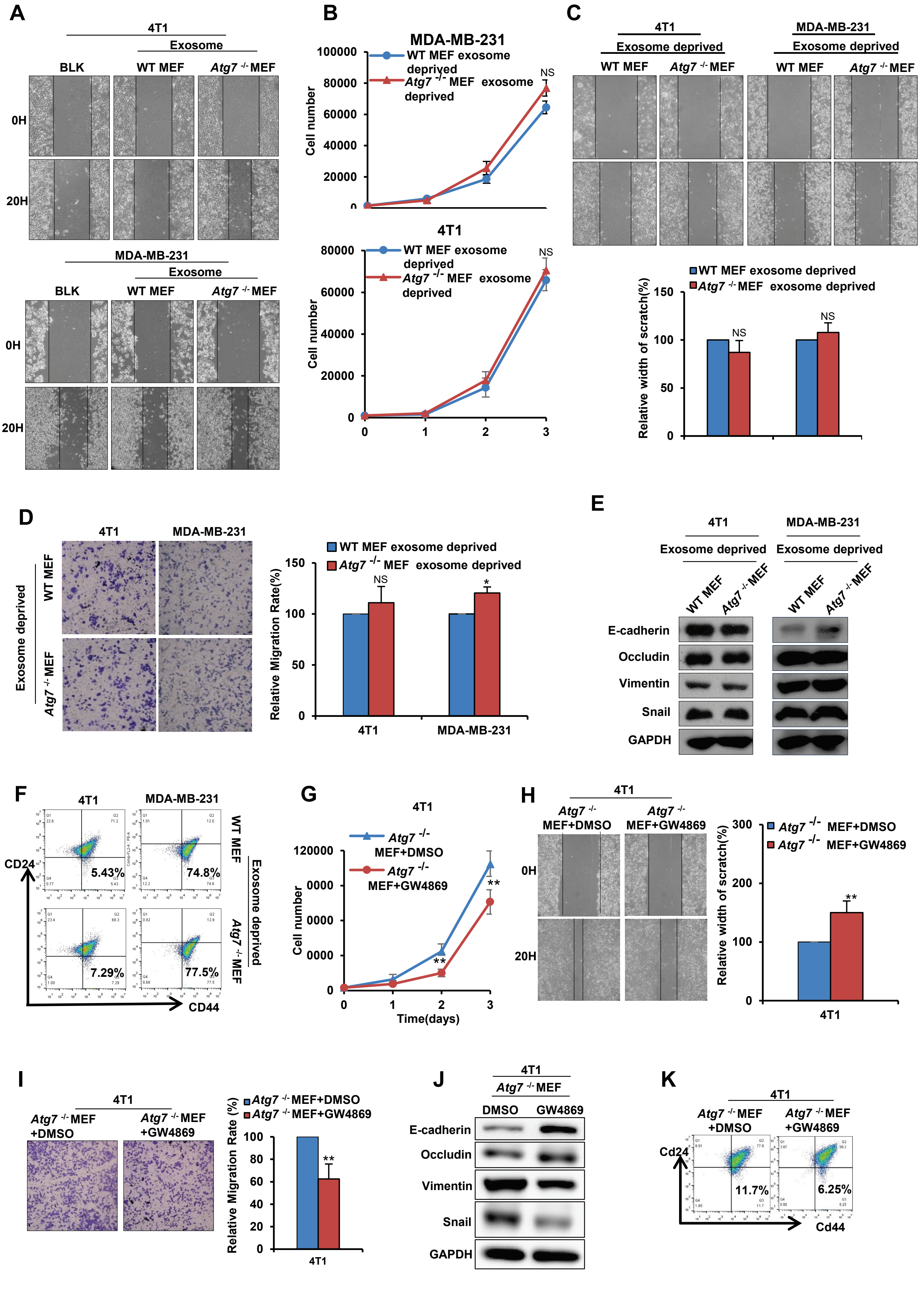

Supplementary Figure 3

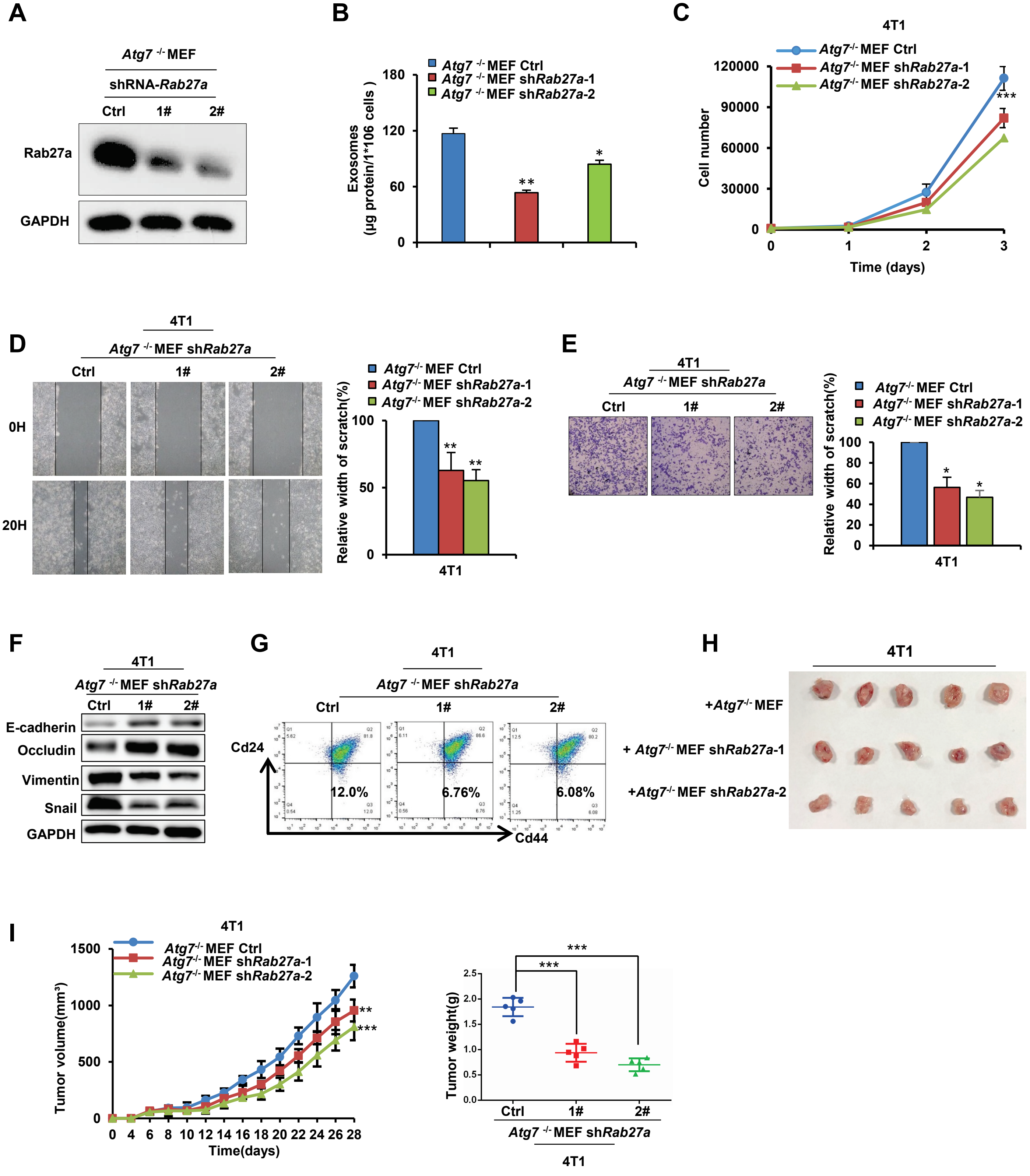

Supplementary Figure 4

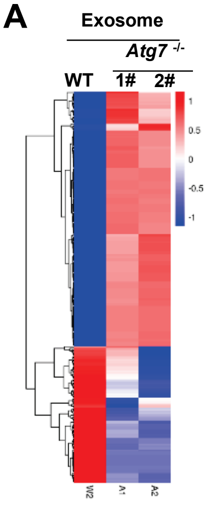

B

miRNA	*Atg7* -/-_readcount	WT_readcount	log2FoldChange	pval	padj
mmu-let-7d-3p	3343.31	1.313413	10.645	1.79E-37	7.15E-35
mmu-miR-484	1408.073	0.437804	10.78	3.27E-33	6.52E-31
mmu-miR-99b-3p	999.0938	0.729674	9.7697	8.72E-31	1.16E-28
novel_41	2605.374	0	13.219	1.40E-30	1.39E-28
mmu-miR-501-3p	640.8591	0.145935	10.651	1.10E-24	8.75E-23
mmu-miR-351-5p	701.6049	0	11.552	1.26E-21	6.27E-20
novel_245	871.1788	5.691457	6.9409	1.05E-20	4.66E-19

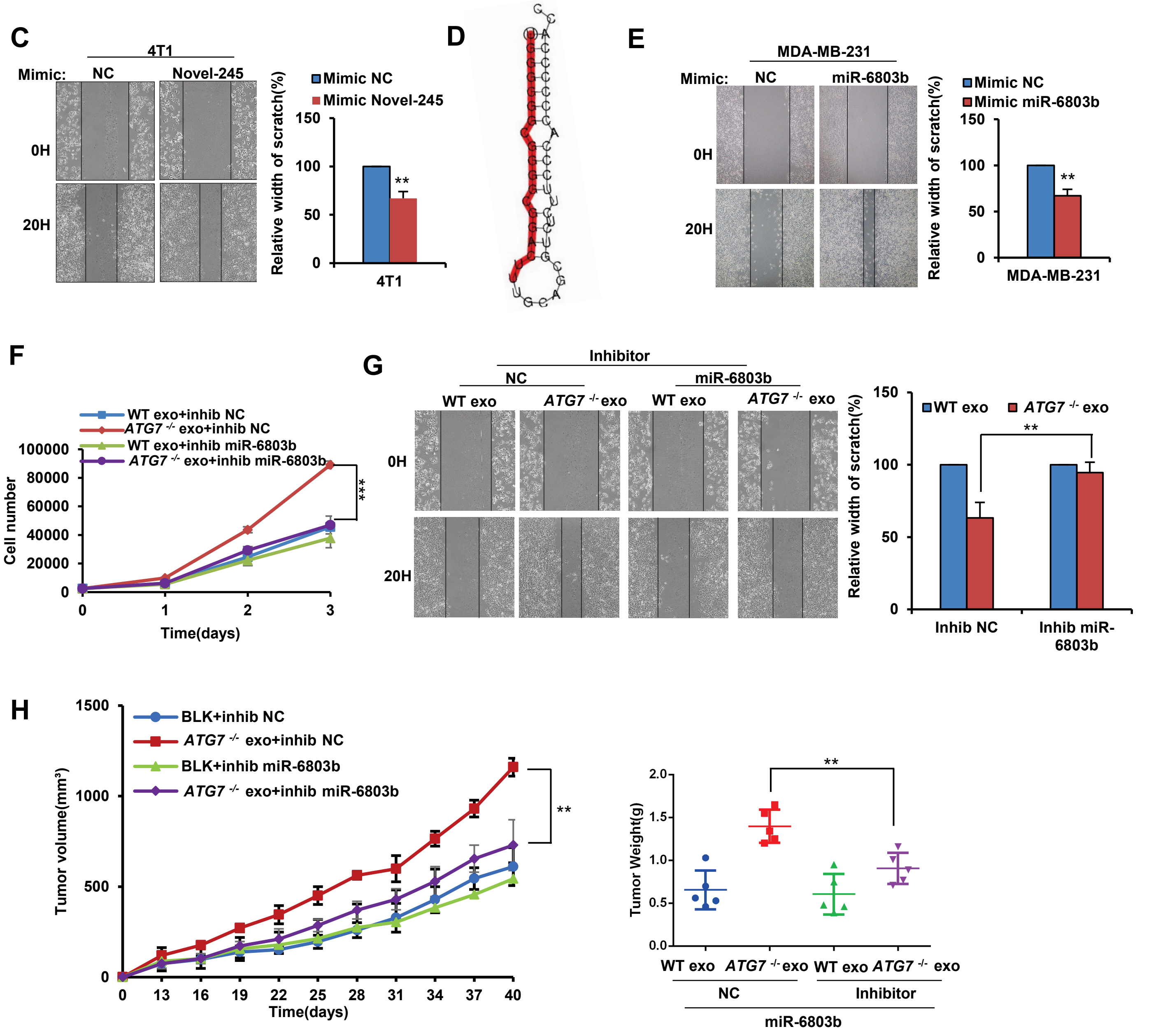

Supplementary Figure 5

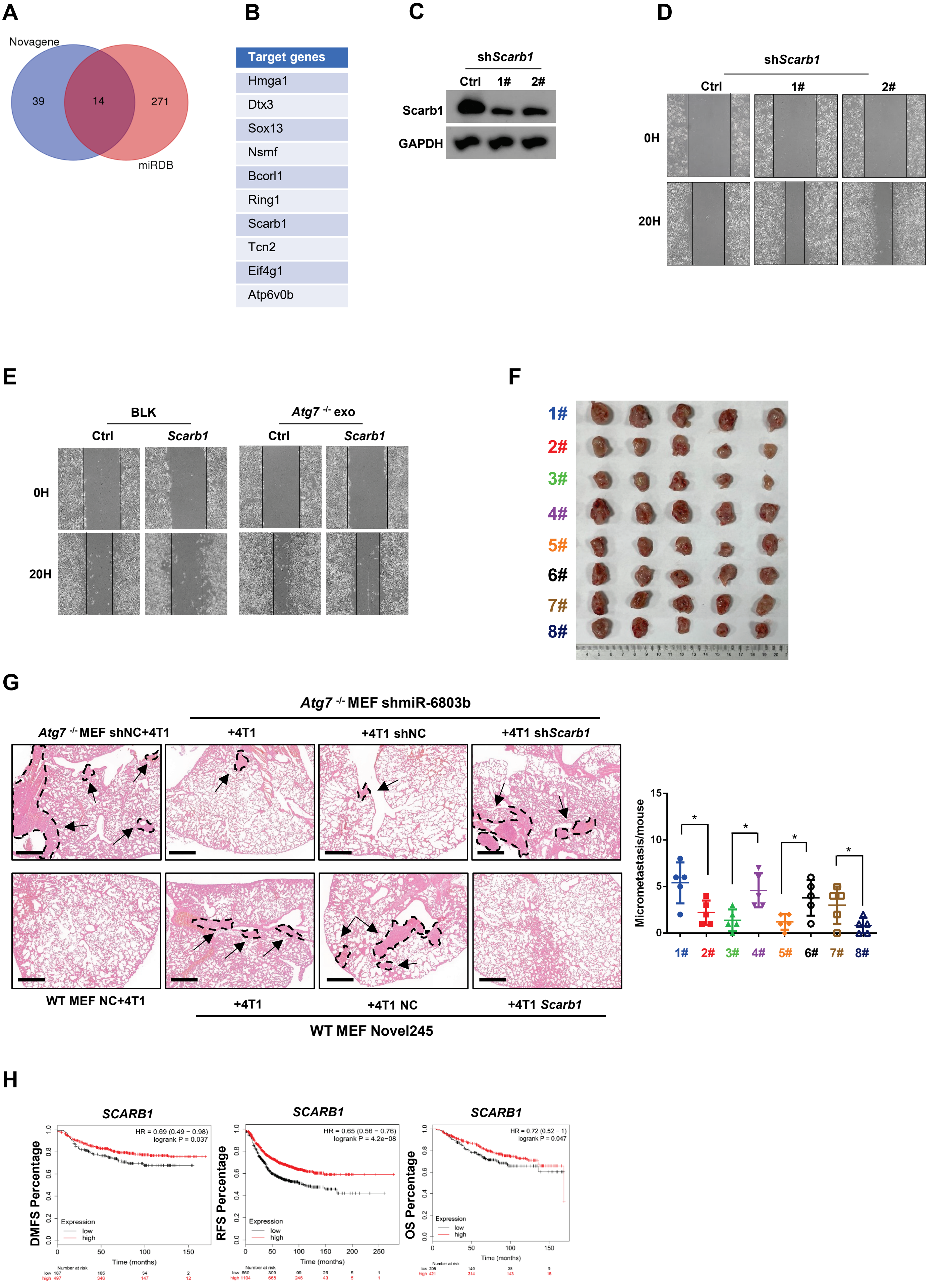

Supplementary Figure 6

A

Number	Pathologic types	Gender	Age	T	N	M	AJCC Stage	Grade	Her-2	LVI	ER	PR	Ki67
1	Hyperplasia	Female	51										
2	Invasive breast carcinoma	Female	48	1a	0	x	1A	Ⅱ	(+)	(+)	(+)	(+)	20%
3	Invasive breast carcinoma	Female	73	1a	0	x	1A	Ⅱ	(-)	(-)	(+)	(+)	10%
4	Invasive breast carcinoma	Female	63	1c	1a	x	2A	Ⅱ	(-)	(+)	(+)	(+)	40%
5	Invasive breast carcinoma(TNBC)	Female	55	2	0	x	2A	Ⅲ	(-)	(-)	(-)	(-)	60%
6	Invasive breast carcinoma	Female	52	2	0	x	2A	Ⅲ	(+)	(+)	(-)	(-)	50%

B

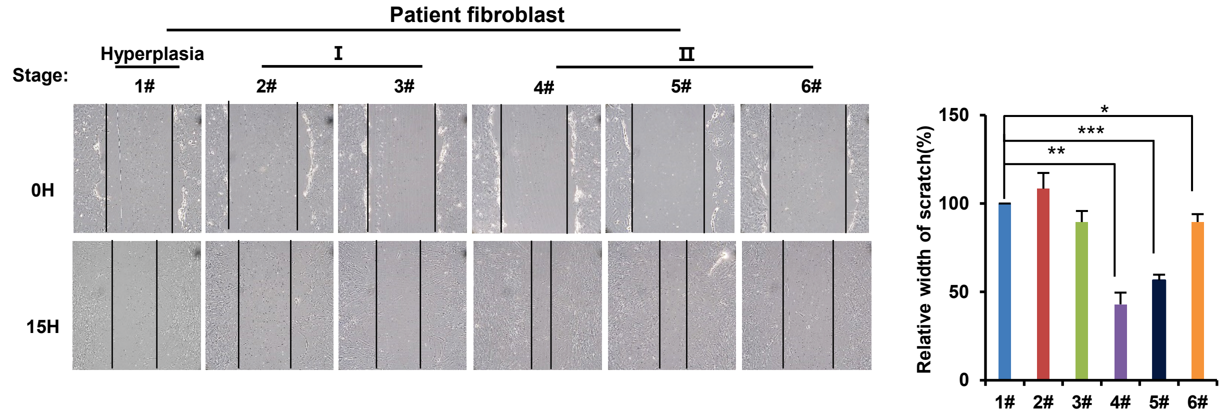

C

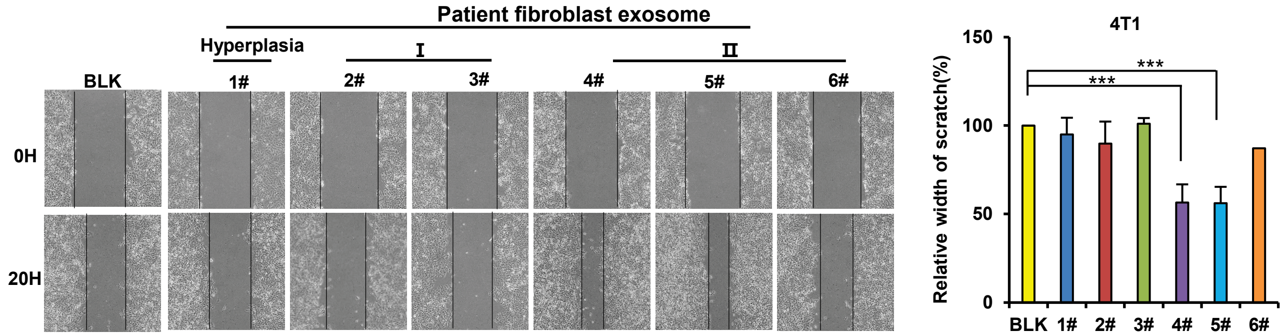

D

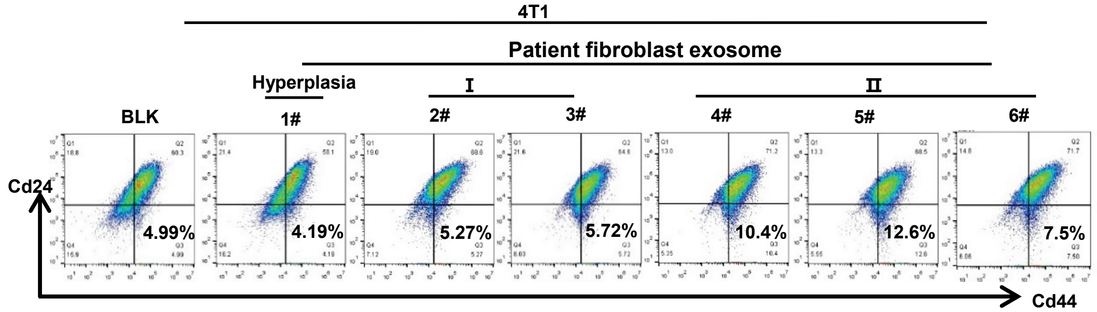

Supplementary Figure 7
