# Supplementary material for: ATG7-deficient fibroblast promotes breast cancer progression via exosome-mediated downregulation of SCARB1
Source: Cell Death Dis. 2025 Jul 24;16(1):556. doi: 10.1038/s41419-025-07885-6 (PMC12289893; doi:10.1038/s41419-025-07885-6)
Supplement: Supplementary file 2 — Supplementary Figure Legends [file 41419_2025_7885_MOESM2_ESM.docx]

**Supplementary Figure Legends：**

**Supplementary Figure Legends 1.**

1. IHC analysis of WT and *Atg7*^-/-^ HFF-1 for the expression of ATG7. Scale bar, 100 μm.
2. Tissue array analysis of 140 breast cancer samples for the expression of ATG7.
3. The three on the left: Survival analysis of the relationship between the OS, DMFS and RFS time of breast cancer patients and the expression of ATG7 based on Kaplan‒Meier plotter online web tool. The one on the far right: Survival analysis of the relationship between the OS time of breast cancer patients and the expression of ATG7 based on XENA online tools. The median expression was used as the cut-off. *P*-value was determined by the log-rank test (HR: hazard ratio) in the analyses.
4. ATG7 expression levels in metastasis tissues or primary tissues of breast cancer from the GEO database (GSE100534). The measure unit of expression is log2-transformed normalized expression levels from the microarray results.
5. Xenograft assays of 4T1-Luc cells or MDA-MB-231-Luc cells mixed with indicated MEFs were performed on BALB/C or BALB/C-NU mice. Representative tumors were shown.
6. Xenograft assays of 4T1-Luc cells mixed with the indicated MEFs were performed on BALB/c mice. Tumor growth curves are shown.
7. Scatterplots of the individual weights of tumors.
8. Xenograft tumors of 4T1-Luc were quantified using bioluminescence imaging after 28 days of subcutaneous injection. Representative in vivo bioluminescent images are shown.
9. Metastatic colonization of 4T1-Luc cells was quantified using bioluminescence imaging after 28 days of subcutaneous injection. Representative in vivo bioluminescent images are shown.
10. Left: Representative H&E-stained sections of lung 4T1-Luc xenograft mice. Metastases are indicated by the arrows. Right: Quantification of metastatic colonization in the lungs.

The experiments were performed at least in triplicate, and the results are presented as the mean ± s.d. The data were analyzed by Student’s t-test (*p < 0.05; **p < 0.01; ***p < 0.001).

**Supplementary Figure Legends 2.**

1. The growth numbers of Ctrl and *Atg7* re-expressed *Atg7*^-/-^ MEFs that were recorded at 1 day, 2 days, 3 days.
2. Scratch assays of Ctrl and *Atg7* re-expressed *Atg7*^-/-^ MEFs.
3. Western blot analysis of ATG7, ACTA2, Vimentin, PDGFR-α, FAP protein levels in Ctrl and *Atg7* re-expressed *Atg7*^-/-^ MEFs.
4. Collagen contraction assay of Ctrl and *Atg7* re-expressed *Atg7*^-/-^ MEFs. Representative images of three replicates of each group at 3 days are shown.
5. Scratch assays of 4T1 and MDA-MB-231 cells treated with the indicated CM for 20h. Representative images are shown.
6. Growth numbers of T47D and MDA-MB-468 cells were recorded after indicated CM treatment for 1 day, 2 days, 3 days and 4 days.
7. The viability of T47D and MDA-MB-468 cells was assessed by the Plate Colony Formation assay after indicated CM treatment for 7 days.
8. Scratch assays of T47D and MDA-MB-468 cells treated with the indicated CM for 36h. Representative images and statistical data are shown.
9. Transwell assays of T47D and MDA-MB-468 cells treated with the indicated CM for 36h. Representative images and statistical data are shown.
10. Growth numbers of MDA-MB-231 cells were recorded after the indicated CM treatment for 1 day, 2 days and 3 days.
11. Western blot analysis of E-cadherin, Vimentin, Occludin and Snail protein levels in the indicated CM-treated MDA-MB-231 cells. GAPDH was used as a loading control.
12. Transwell assays of MDA-MB-231 cells treated with the indicated CM for 20 h. Representative images are shown, and the migrated cells were counted.
13. Flow cytometry analysis of the cancer stem-like cell population (CD44^high^/CD24^low^) in the indicated CM-treated MDA-MB-231 cells.
14. Protein quantification in exosomes isolated from the CM of WT MEFs and *Atg7*^-/-^ MEFs by BCA assay.

The experiments were performed at least in triplicate, and the results are presented as the mean ± s.d. The data were analyzed by Student’s t-test (*p < 0.05; **p < 0.01; ***p < 0.001).

**Supplementary Figure Legends 3.**

1. Scratch assays of 4T1 and MDA-MB-231 cells treated with the indicated exosomes for 20h. Representative images are shown.
2. Growth numbers of 4T1 and MDA-MB-231 cells were recorded after indicated CM treatment for 1 day, 2 days and 3 days.
3. Scratch assays of 4T1 and MDA-MB-231 cells treated with the indicated CM for 20 h. Representative images and statistical data are shown.
4. Transwell assays of 4T1 and MDA-MB-231 cells treated with the indicated CM for 20h. Representative images and statistical data are shown, and the migrated cells were counted.
5. Western blot analysis of E-cadherin, Vimentin, Occludin and Snail protein levels in indicated CM treated 4T1 and MDA-MB-231. GAPDH was used as a loading control.
6. Flow cytometry analysis of the cancer stem–like cell population (CD44^high^/CD24^low^) in indicated CM treated 4T1 and MDA-MB-231.
7. Growth numbers of 4T1 cells were recorded after treatment with the indicated CM (Collected from WT MEFs and *Atg7*^-/-^ MEFs treated with 10 μM GW4869 or DMSO for 2 days) for 1 day, 2 days and 3 days.
8. Scratch assays of 4T1 cells treated with the indicated CM (collected from WT MEFs and *Atg7*^-/-^ MEFs treated with 10 μM GW4869 or DMSO for 2 days) for 20 h. Representative images and statistical data are shown.
9. Transwell assays of 4T1 cells treated with the indicated CM (collected from WT MEFs and *Atg7*^-/-^ MEFs treated with 10 μM GW4869 or DMSO for 2 days) for 20 h. Representative images are shown, and the migrated cells were counted.
10. Western blot analysis of E-cadherin, Vimentin, Occludin and Snail protein levels in the indicated CM (collected from WT MEFs and *Atg7*^-/-^ MEFs treated with 10 μM GW4869 or DMSO for 2 days) treated with 4T1 cells. GAPDH was used as a loading control.
11. Flow cytometry analysis of the cancer stem-like cell population (CD44^high^/CD24^low^) in the indicated CM (collected from WT MEFs and *Atg7*^-/-^ MEFs treated with 10 μM GW4869 or DMSO for 2 days) treated with 4T1 cells.

The experiments were performed at least in triplicate, and the results are presented as the mean ± s.d. The data were analyzed by Student’s t-test (*p < 0.05; **p < 0.01; ***p < 0.001).

**Supplementary Figure Legends 4.**

1. Western blotting analysis of Rab27a protein in shRab27a -expressing *Atg7*^-/-^ MEF and control cells. GAPDH was used as a loading control.
2. Protein quantification in exosomes isolated from the CM of shRab27a -expressing *Atg7*^-/-^ MEF and control cells by BCA assay.
3. Growth numbers of 4T1 cells were recorded after the indicated CM (Collected from shRab27a-expressing *Atg7*^-/-^ MEFs or control cells) treatment for 1 day, 2 days and 3 days.
4. Scratch assays of 4T1 cells treated with the indicated CM (Collected from shRab27a-expressing *Atg7*^-/-^ MEFs or control cells) for 20 h. Representative images and statistical data are shown.
5. Transwell assays of 4T1 cells treated with the indicated CM (Collected from shRab27a-expressing *Atg7*^-/-^ MEFs or control cells) for 20 h. Representative images are shown, and the migrated cells were counted.
6. Western blot analysis of E-cadherin, Vimentin, Occludin and Snail protein levels in the indicated CM (Collected from shRab27a-expressing *Atg7*^-/-^ MEFs or control cells) treated with 4T1. GAPDH was used as a loading control.
7. Flow cytometry analysis of the cancer stem-like cell population (CD44^high^/CD24^low^) in the indicated CM (collected from shRab27a-expressing *Atg7*^-/-^ MEFs or control cells) treated with 4T1 cells.
8. Xenograft assays of 4T1-Luc cells mixed with shrRab27a-expressing *Atg7*^-/-^ MEF or Control cells were performed on BALB/C mice. Representative tumors were shown.
9. Xenograft assays of 4T1-Luc cells mixed with shRab27a-expressing *Atg7*^-/-^ MEFs or control cells were performed in BALB/c mice. Tumor growth curves (left) and scatterplots of the individual weights of tumors (right) are shown.

The experiments were performed at least in triplicate, and the results are presented as the mean ± s.d. The data were analyzed by Student’s t-test (*p < 0.05; **p < 0.01; ***p < 0.001).

**Supplementary Figure Legends 5.**

- 1. Small RNAseq analysis of exosomal miRNAs from WT MEFs and *Atg7-/-* MEFs is presented in a heatmap.
  2. The detailed sequencing information of top 7 significantly differentially expressed miRNAs.
  3. Scratch assays of 4T1 cells treated with the indicated miRNA mimic or control for 20 h. Representative images and Statistical data are shown.
  4. Schematic illustration of the stem‒loop portion of Novel-245.
  5. Scratch assays of MDA-MB-231 cells treated with the indicated miRNA mimic or control for 20 h. Representative images and Statistical data are shown.
  6. Growth numbers of 4T1 cells were recorded after the indicated exosomes or miRNA inhibitor treatment for 1 day, 2 days and 3 days.
  7. Scratch assays of 4T1 cells treated with the indicated exosomes or miRNA inhibitor for 20 h. Statistical data and Representative image are shown.
  8. Xenograft assays of MDA-MB-231-Luc cells in BALB/c-NU mice. *ATG7^-/-^* HFF-1 exosomes or miR-6803b inhibitor are injected into tumors. Tumor growth curves (left) and scatterplots of the individual weights of tumors (right) are shown.

The experiments were performed at least in triplicate, and the results are presented as the mean ± s.d. The data were analyzed by Student’s t-test (*p < 0.05; **p < 0.01; ***p < 0.001).

**Supplementary Figure Legends 6.**

- 1. Target gene prediction of miR-6803b with two bioinformatics tools.
  2. Selected top 10 target genes of miR-6803b.
  3. Western blotting analysis of Scarb1 protein in shScarb1-expressing 4T1 cells and control cells. GAPDH was used as a loading control.
  4. Scratch assays of shScarb1-expressing 4T1 cells and control cells. Representative images are shown.
  5. Scratch assays of shScarb1-overexpressing 4T1 cells and control cells treated with the indicated exosomes for 20 h. Representative images are shown.
  6. Xenograft assays of 4T1-Luc cells (infected with lentiviruses carrying the SOD1-overexpressing or shScarb1) mixed with WT MEF (infected with lentiviruses carrying the miR-6803b-overexpressing) or *Atg7*^-/-^ MEF (infected with lentiviruses carrying the shNovel-245) were performed on BALB/C mice. Representative tumors were shown.
  7. Left: Representative H&E-stained sections of lungs 4T1-Luc xenograft mice, metastases are indicated by the arrows. Right: Quantification of metastatic colonization in the lungs.
  8. Correlation of *SCARB1* expression with OS, DMFS and RFS using Kaplan‒Meier plotter online web tool. Statistical significance was estimated using a log-rank test.

The experiments were performed at least in triplicate, and the results are presented as the mean ± s.d. The data were analyzed by Student’s t-test (*p < 0.05; **p < 0.01; ***p < 0.001).

**Supplementary Figure Legends 7.**

- 1. The clinicopathological features of 6 patients.
  2. Scratch assays of fibroblasts from breast cancer patient samples. Representative images and Statistical data are shown.
  3. Scratch assays of 4T1 cells treated with the indicated exosomes (isolated from breast cancer patient fibroblasts) for 20h. Representative images and Statistical data are shown.
  4. Flow cytometry analysis of the cancer stem-like cell population (CD44^high^/CD24^low^) in the indicated exosomes (isolated from breast cancer patient fibroblasts) treated with 4T1 cells.

The experiments were performed at least in triplicate, and the results are presented as the mean ± s.d. The data were analyzed by Student’s t-test (*p < 0.05; **p < 0.01; ***p < 0.001).
