# Supplementary material for: ATG7-deficient fibroblast promotes breast cancer progression via exosome-mediated downregulation of SCARB1
Source: Cell Death Dis. 2025 Jul 24;16(1):556. doi: 10.1038/s41419-025-07885-6 (PMC12289893; doi:10.1038/s41419-025-07885-6)
Supplement: Supplementary file 4 — Supplementary Materials and Methods [file 41419_2025_7885_MOESM4_ESM.docx]

**Supplementary Materials and Methods**

**FACS analysis**

For cell stemness detection, CM-treated cells were washed with PBS and stained with CD24-PE (5554728, BD, NY, USA) and CD44-FITC (555478, BD) for 15 min. The stained cells were then assessed by flow cytometry and analyzed by FlowJo (version X.0.7).

ALDH detection was performed according to the kit instructions.

**Gel electrophoresis and immunoblotting**

Cells were harvested and lysed on ice in 1% SDS. Then, the cell lysates were heated at 98 °C for 20 min, after which the supernatants were collected by high-speed centrifugation. A bicinchoninic acid (BCA) assay was performed to measure the protein concentrations. Approximately 10–50 μg of equal amounts of protein were resolved by SDS‒PAGE, after which the membranes were blocked in TBST solution supplemented with 5% milk (Bio-Rad, USA) for 2 h and subsequently incubated with specific antibodies. Then, the membranes were washed with TBST and incubated with HRP-conjugated secondary antibodies for 2 h at room temperature. After incubation with horseradish peroxidase-conjugated secondary antibodies, proteins were detected using Immobilon™ Western Chemiluminescent HRP Substrate (Millipore). The images of protein bands obtained from Western blotting were analyzed using ImageJ software (National Institutes of Health, Bethesda, MD).

**Migration assay**

For the scratch assay, cells were seeded into 12-well plates, and a scratch was created by manually drawing a gap with a pipette tip. The floating cells were gently rinsed away with PBS, and then Serum-free medium, Serum-free CM (Supernatants: Serum-free medium at a 1:1 ratio) or exosomes resuspended in Serum-free medium were added. The ability of cells to migrate into the cleared section was recorded at 0 h, 20 h and 36h.

For the Transwell assay, cells were plated in 24-well transwell plates with inserts (353097, Falcon). Then, CM or exosomes were added to the lower chamber of 24-well plates. After 24 h, the cell inserts were fixed and stained according to the manufacturer’s protocols. Three randomly representative fields were photographed, and the number of migrated cells per field was counted.

**RNA interference, plasmid construction, lentivirus packaging, and infection**

The plasmids encoding *Scarb1, Luciferase* and the 3'UTR of *Ring1, Hmga1, Scarb1, Dtx3 Sox13* were cloned into pHAGE-CMV-MCS-PGK or pMIR-REPORT. The empty vector was used as a negative control. Site-directed mutagenesis of the *Scarb1*-3'UTR *and Dtx3*-3'UTR was performed using a Mut Express II Fast Mutagenesis Kit (C215-01/02, Vazyme, China). Lentiviral supernatants were generated by transfecting HEK-293T cells with the pHAGE-puro-Scarb1 or pLV3-puro-Atg7 plasmid along with the packaging plasmid (psPAX2 and pMD2.G). Then, the harvested viral supernatants were used to infect cancer cells, and puromycin was added to establish stable cells.

For short hairpin RNA (shRNA)-expressing constructs, short hairpin RNAs targeting *Rab27a* and *Scarb1* were inserted into the PLKO.1 plasmid, and the empty vector was used as a negative control. The methods for generating lentiviruses and screening stable cell lines are consistent with the above descriptions.

Inhibitors of miR-6803b was synthesized and purchased by AuGCT Biotech Co., Ltd (Wuhan, China). The sequence of the inhibitor was 5'- A*A*G*UCCGCCCCGCCCC*C*C*A-3', the symbol * indicates phosphorothioate diester bond which is used for stability and specificity. The inhibitor was delivered via Lipo3000 and treated cells for 24 h.

**Immunofluorescence**

Fibroblasts were seeded on coverslips, fixed with 4% paraformaldehyde for 20 min, blocked with PBS supplemented with 1% BSA and permeabilized with 0.1% Triton-100. After incubation with primary antibody and Alexa-488-conjugated secondary antibodies, representative images were obtained using confocal microscopy (SP8, Leica, Wetzlar, Germany).

**Luciferase reporter assay**

Luciferase activities were detected using the Dual Luciferase kit (Promega, USA) according to the manufacturer’s recommendations. The results were normalized to the constitutively expressed Renilla luciferase.

**Microarray analysis of exosomal miRNAs**

Exosomal miRNA microarray analysis was performed at Novogene Biotechnology Corporation (Beijing, China), and the data were processed using the Bioconductor package of R tools. After quantile normalization, fold changes were calculated on the basis of the mean values of the replicates.
