# Supplementary material for: ATG7-deficient fibroblast promotes breast cancer progression via exosome-mediated downregulation of SCARB1
Source: Cell Death Dis. 2025 Jul 24;16(1):556. doi: 10.1038/s41419-025-07885-6 (PMC12289893; doi:10.1038/s41419-025-07885-6)

Figure 2C

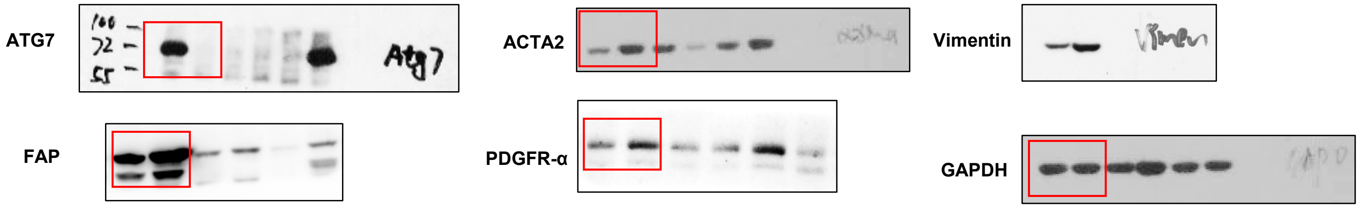

Figure 3E

4T1

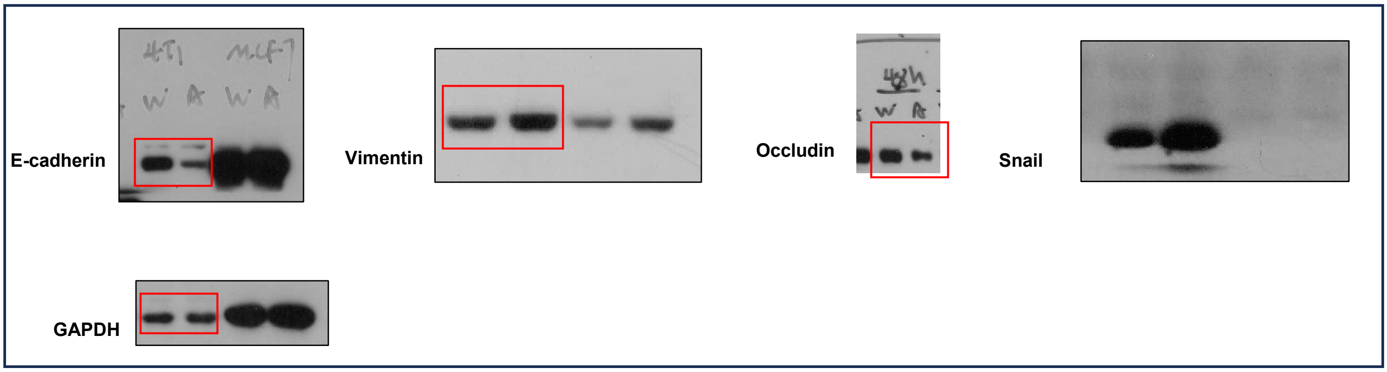

MDA-MB-231

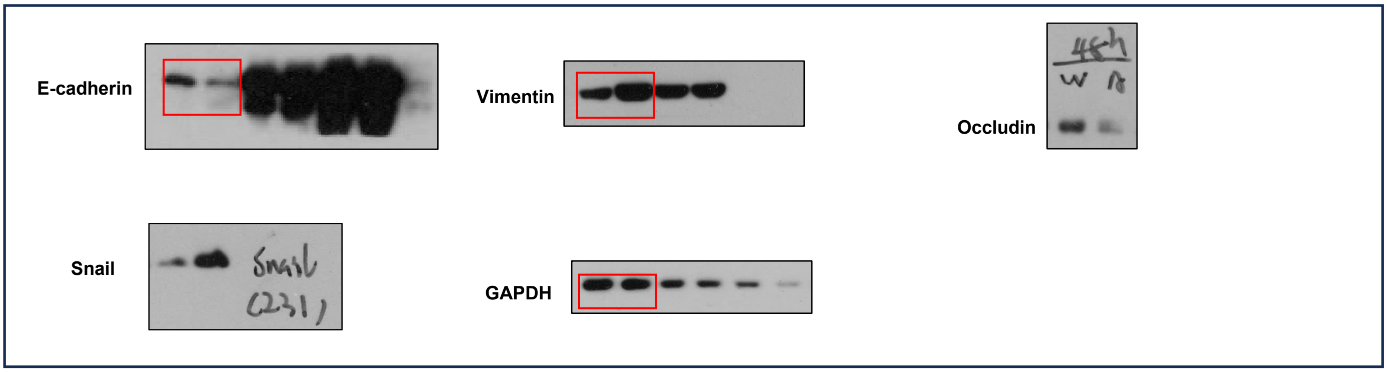

Figure 4C

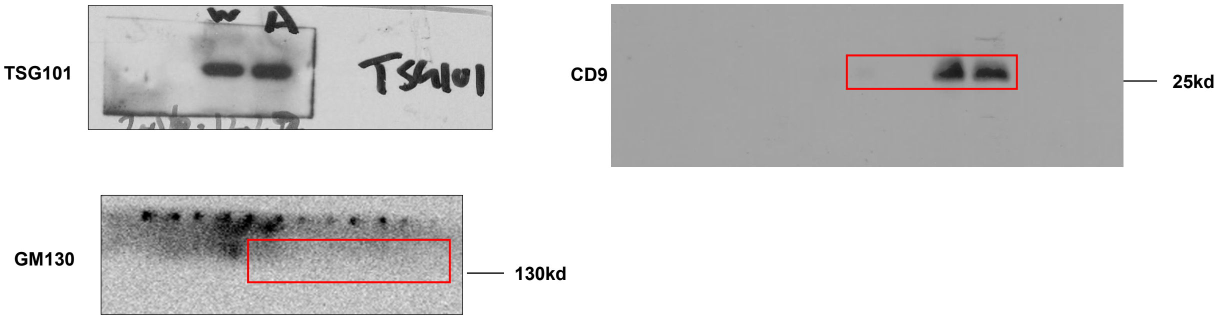

Figure 4H

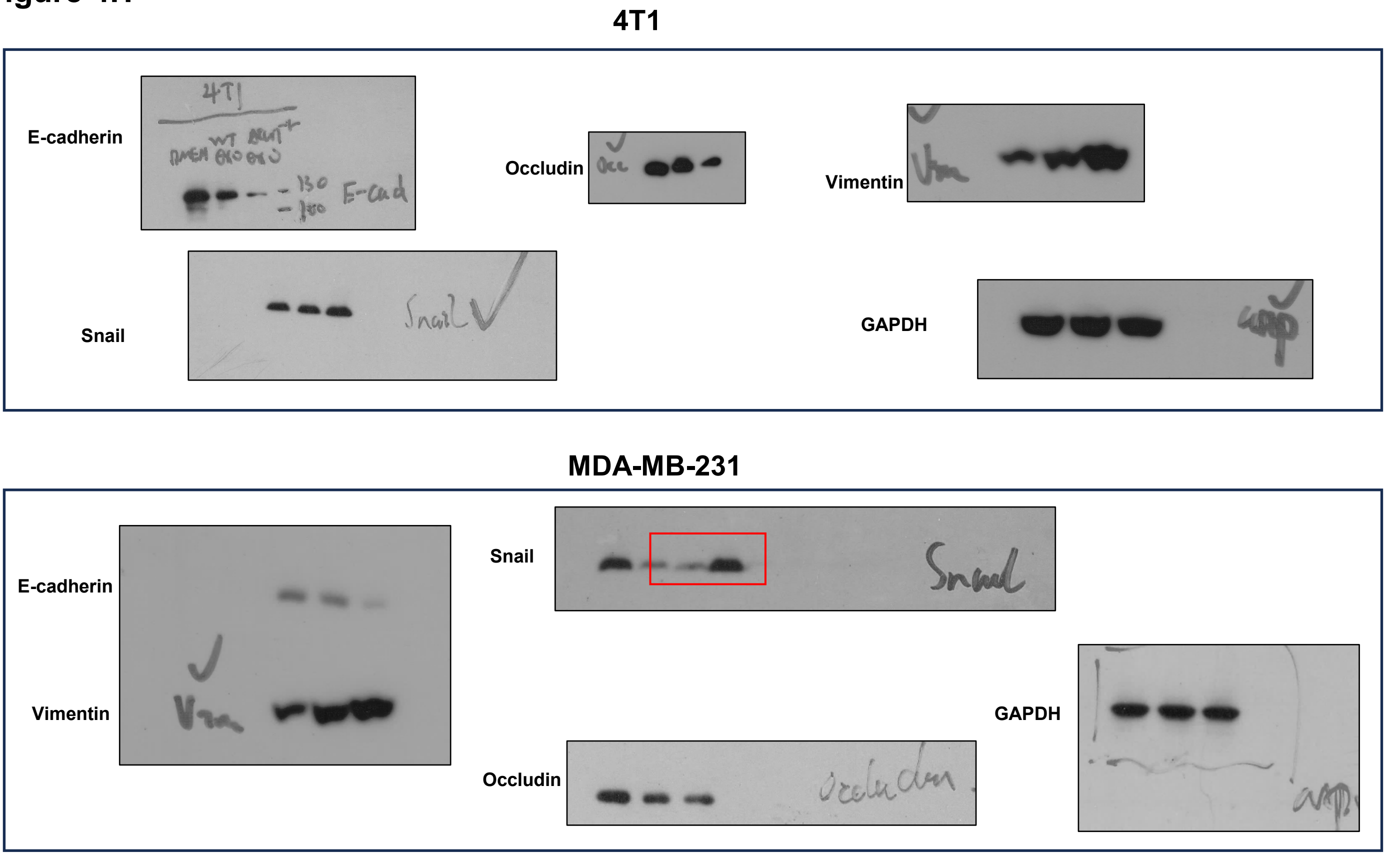

Figure 5A

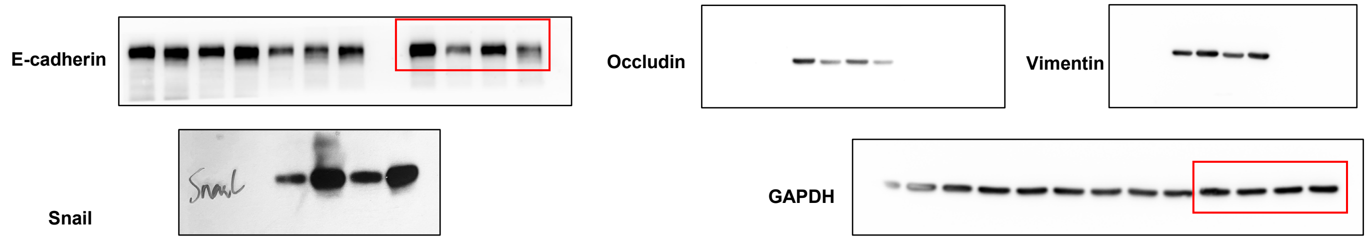

Figure 5D

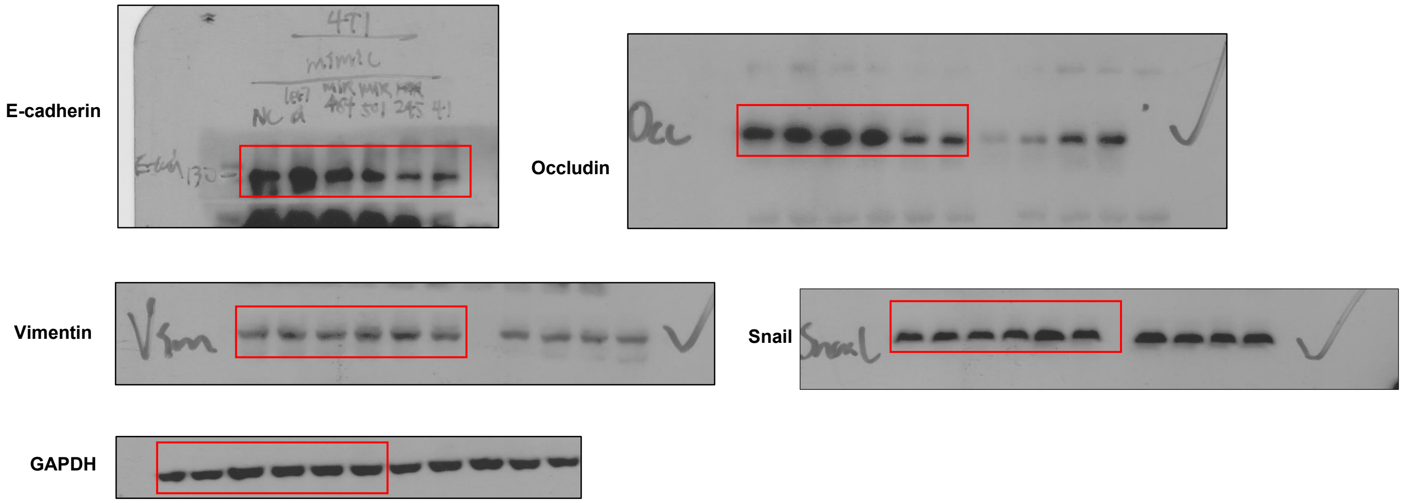

Figure 5O

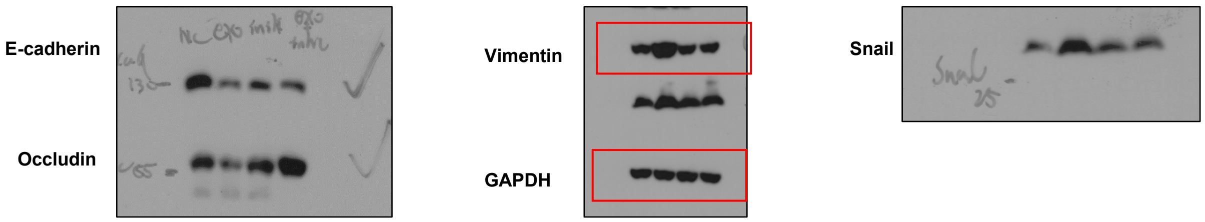

Figure 6G

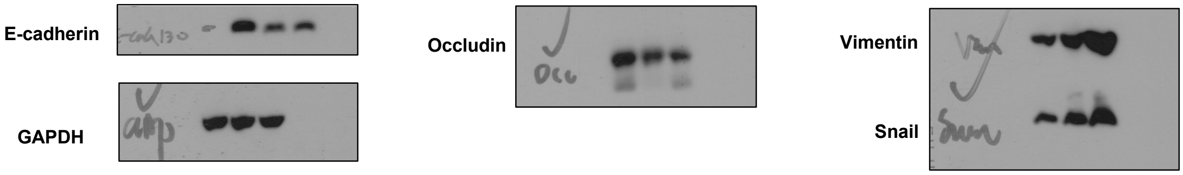

Figure 7A

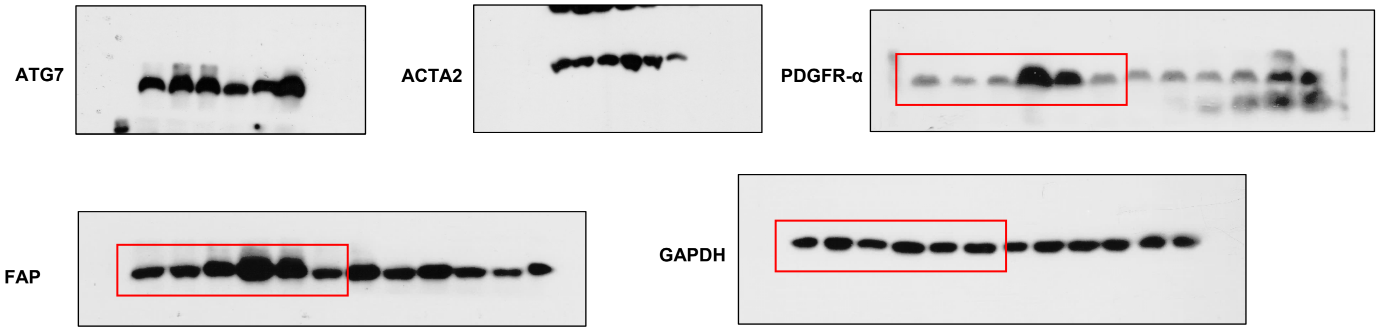

Figure 7F

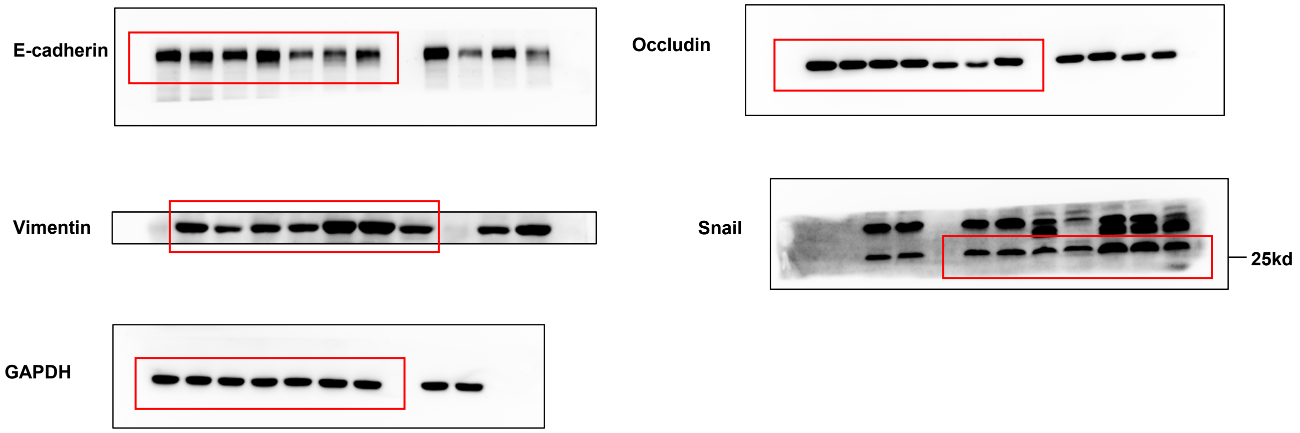

Figure 7G

2#-sh-ATG7

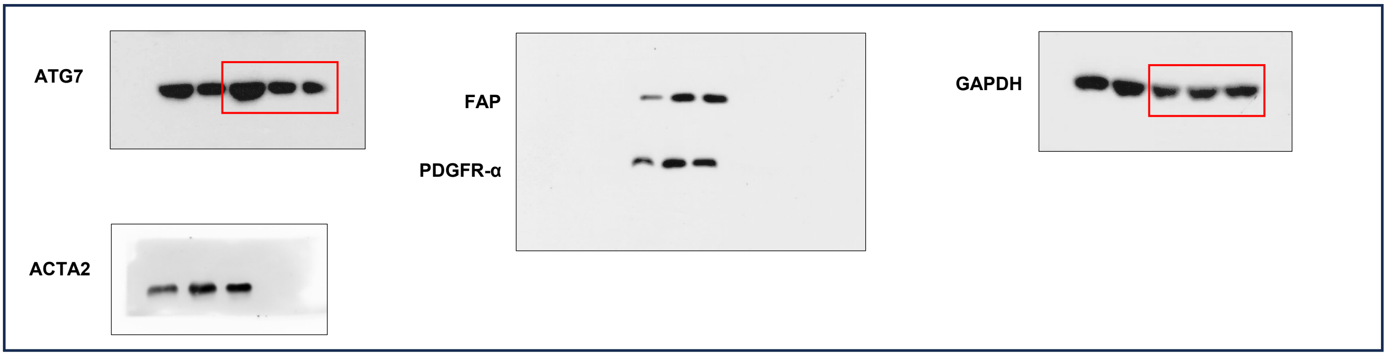

4#-OE

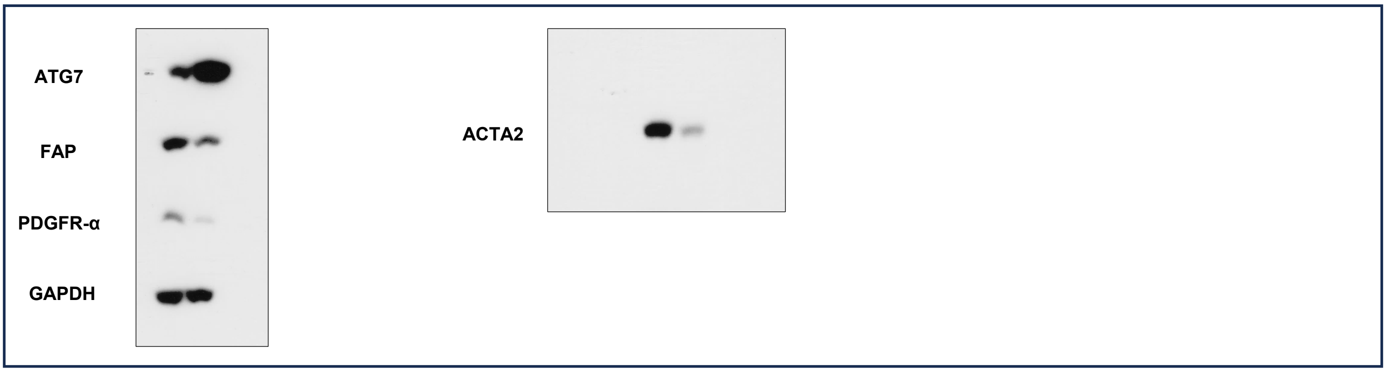

Figure 7J

2# exo treated MDA-MB-231

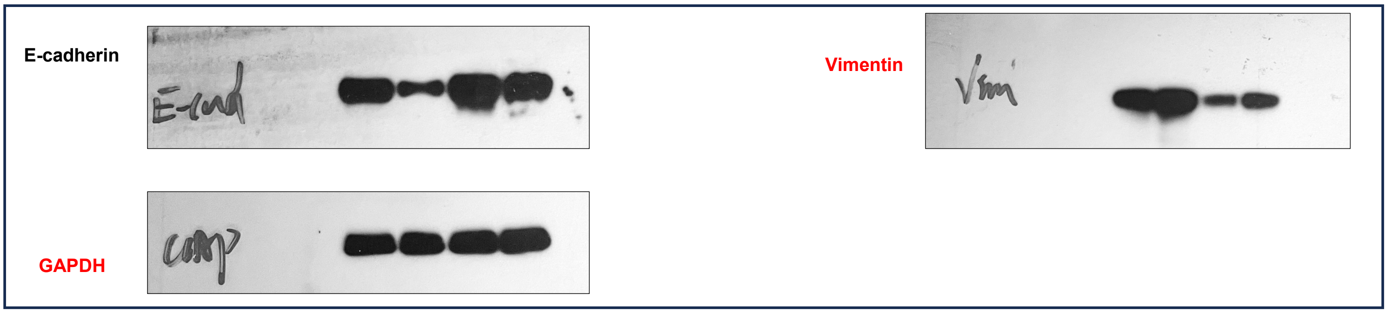

4# exo treated MDA-MB-231

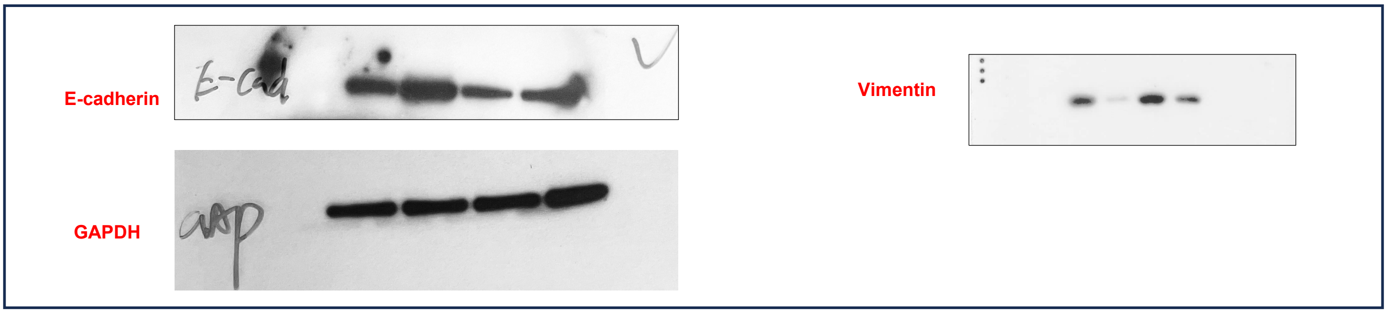

Supplementary Figure 2C

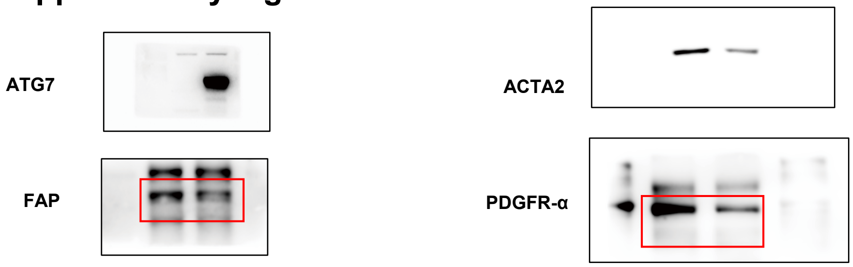

Supplementary Figure 2K

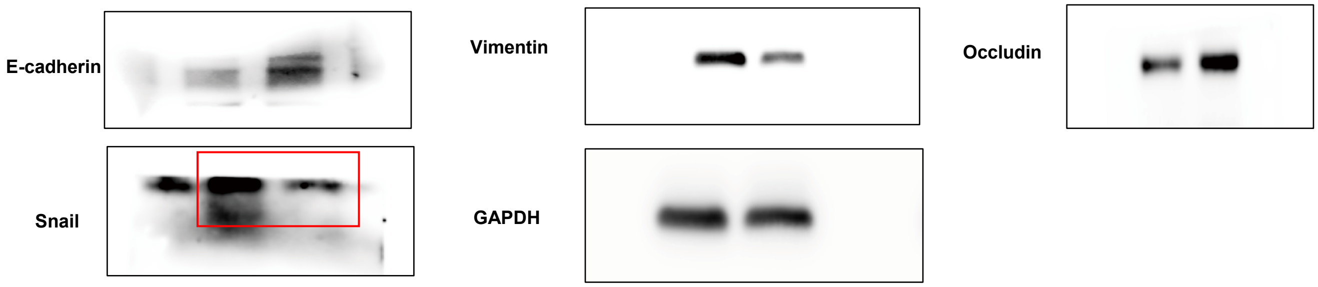

Supplementary Figure 3E

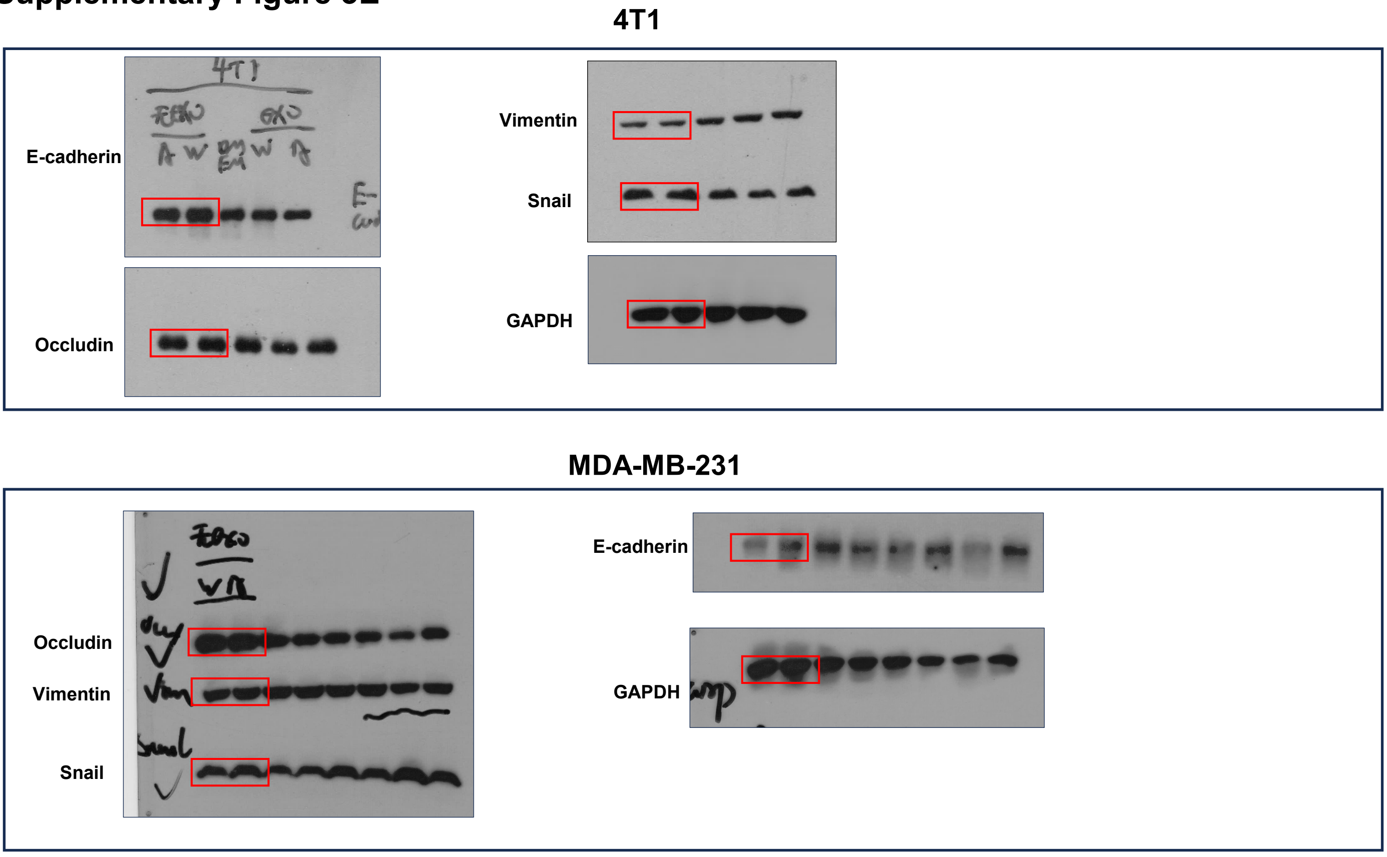

Supplementary Figure 3J

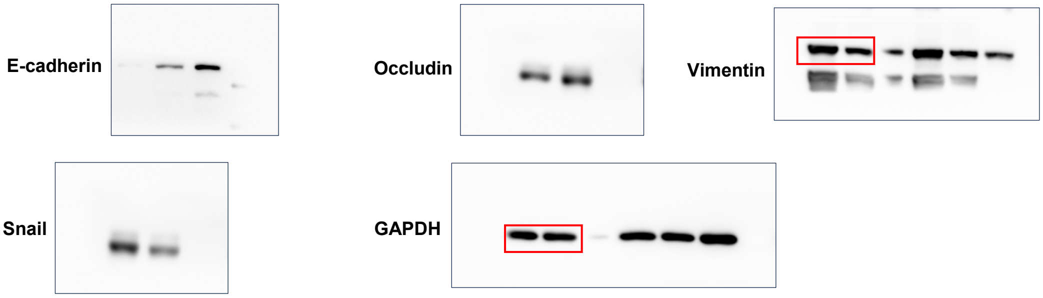

Supplementary Figure 4A

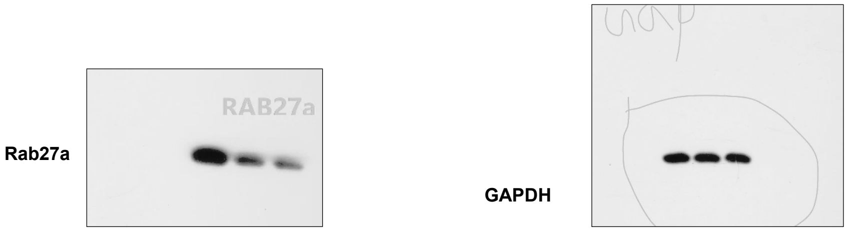

Supplementary Figure 4F

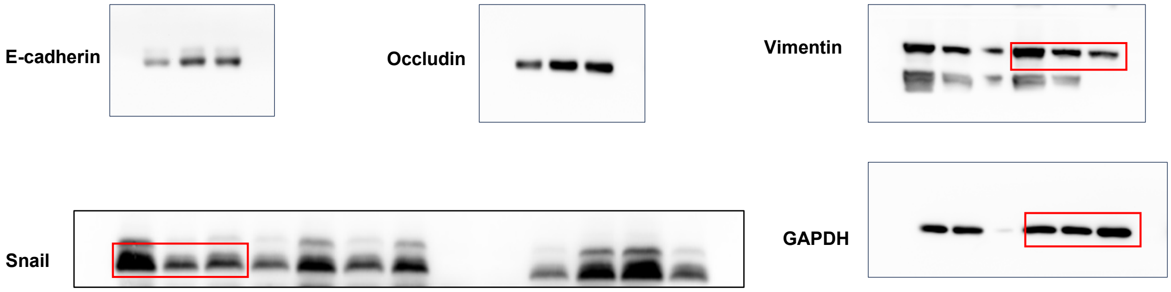

Supplementary Figure 6C

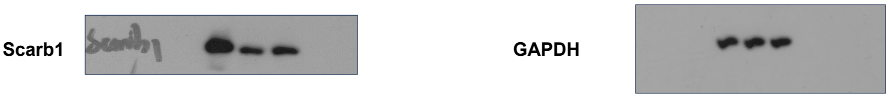

Supplement: Supplementary file 5 — Full and uncropped western blots [file 41419_2025_7885_MOESM5_ESM.pdf]
